# Cytotoxic compounds from *Laurencia pacifica*

**DOI:** 10.1186/s13588-014-0008-8

**Published:** 2014-09-20

**Authors:** Diana A Zaleta-Pinet, Ian P Holland, Mauricio Muñoz-Ochoa, J Ivan Murillo-Alvarez, Jennette A Sakoff, Ian A van Altena, Adam McCluskey

**Affiliations:** 1 Chemistry, School of Environmental and Life Science, The University of Newcastle, University Drive, Callaghan 2308, NSW, Australia; 2 Development Technology Department, Interdisciplinary Centre of Marine Sciences, National Technological Institute, La Paz, Mexico; 3 Department of Medical Oncology, Calvary Mater Newcastle Hospital, Waratah 2298, NSW, Australia

**Keywords:** Laurencia pacifica, Algae, Sesquiterpenes, Anti-cancer, Cytotoxicity

## Abstract

**Background:**

The current investigation sought to explore the nature of the secondary metabolites in the algae, *Laurencia pacifica*.

**Results:**

This report details the first isolation of the sesquiterpenes isoaplysin (**1**), isolaurenisol (**2**), debromoisolaurinterol (**3**), debromoaplysinol (**4**), laur-11-en-10-ol (**5**), 10?-hydroxyldebromoepiaplysin (**6**), and the previously unknown 10-bromo-3,7,11,11-tetramethylspiro[5.5]undeca-1,7-dien-3-ol (**7**) from the algae, *Laurencia pacifica*. Isoaplysin (**1**) and debromoaplysinol (**4**) showed promising levels of growth inhibition against a panel cancer-derived cell lines of colon (HT29), glioblastoma (U87, SJ-G2), breast (MCF-7), ovarian (A2780), lung (H460), skin (A431), prostate (Du145), neuroblastoma (BE2-C), pancreas (MIA), murine glioblastoma (SMA) origin with average GI_50_ values of 23 and 14 ?M.

**Conclusions:**

Isoaplysin (**1**) and debromoaplysinol (**4**) were up to fourfold more potent in cancer-derived cell populations than in non-tumor-derived normal cells (MCF10A). These analogues are promising candidates for anticancer drug development.

**Graphical Abstract:**
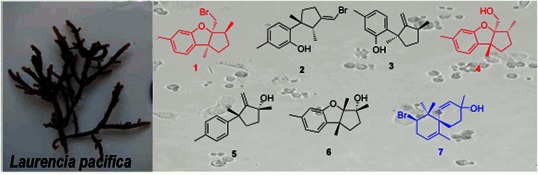
?

## Findings

### Introduction

Natural products with their high fraction *sp*^*3*^ content (F*sp*^3^) represent a significant proportion of all clinical drugs [1]. Of the 1,355 new entities introduced as therapeutics between 1981 and 2010, 71% were natural products or natural product derived [2]. A high F*sp*^3^ content imbues natural products with defined three-dimensional geometry that allows for high levels of interaction with a wide range of biological targets. A significant number of natural products adhere to the `rule of five¿ and thus present high levels of drug-like character [3],[4]. Natural products also afford access to a wide range of novel chemical motifs accessing new chemical space in the drug design and development arena. This has led to the ongoing interest in accessing natural product secondary metabolites (in particular) with their high chemical diversity and biological specificity, making them a favorable source of lead compounds for drug discovery and development [5],[6].

Recently, we turned our attention to marine natural products as a potential source for new lead compounds. In this area, we have identified a small family of cytotoxic steroids from an Australian sponge *Psammoclema* sp. [7], and antimalarial, antialgal, antitubercular, antibacterial, antiphotosynthetic, and antifouling activity of diterpene and diterpene isonitriles from the tropical marine sponge *Cymbastela hooperi*[8]. In this present study, we examined the cytotoxicity of extracts obtained from *Laurencia pacifica* algae collected in the pacific coast of the Baja California Peninsula, Mexico.

The genus *Laurencia* typically inhabits the world's tropical oceans and has been responsible for approximately half of all the reported compounds from red algae. This genus is considered an important producer of halogenated sesquiterpenes, diterpenes, and acetogenins [9]-[11]. Biological activities of the *Laurencia* family range from antipredatory [12], antifungal [13], antibacterial [14]-[16], to anticancer [17]-[19]. Secondary metabolites reported from *L. pacifica* include ?-bisabolene, bromocuparane, laurinterol, debromolauriterol, isolaurinterol, aplysin, debromoaplysin, 10-bromo-?-chamigrene, prepacifenol, pacifenol, pacifidine, and kylinone [10].

### Results and discussion

The algae, *L. pacifica* was collected from the Baja California Peninsula, Mexico. The ethanol extracts were examined for the potential presence of cytotoxic compounds. Cytotoxicity screening was conducted against a panel of cancer cell lines of colon (HT29), glioblastoma (U87, SJ-G2), breast (MCF-7), ovarian (A2780), lung (H460), skin (A431), prostate (Du145), neuroblastoma (BE2-C), pancreas (MIA), murine glioblastoma (SMA) origin, and a normal line of breast cells (MCF10A) [20]. The preliminary screening showed sufficient promise to embark on an isolation program (data not shown).

**Figure 1:**
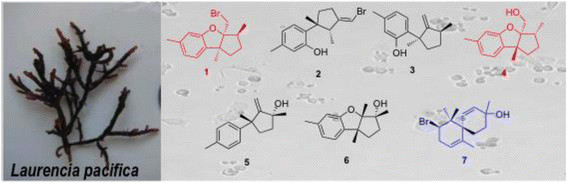
**Chemical structures of the known sesquiterpenes isolated from*Laurencia pacifica*in this work.** Isoaplysin **(1)**, isolaurenisol **(2)**, debromoisolaurinterol **(3)**, debromoaplysinol **(4)**, laur-11-en-10-ol **(5)**, and 10?-hydroxyldebromoepiaplysin **(6)**.

**Figure 2:**
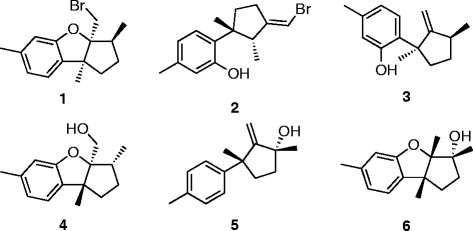
Important HMBC (blue arrows) and COSY (bold bonds) correlations for 7, and the chemical structure of the related 10-bromo-7,8-epoxychamigr-1-en-3-ol (8).

Bioassay-guided fractionation (normal phase chromatography) of the *L. pacifica* ethanolic extracts (see Additional file 1) resulted in the isolation of seven sesquiterpenes: isoaplysin (**1**) [21],[22], isolaurenisol (**2**) [13],[22], debromoisolaurinterol (**3**) [23], debromoaplysinol (**4**) [10],[13],[21], laur-11-en-10-ol (**5**) [13], 10?-hydroxyldebromoepiaplysin (**6**) [13] (Figure 1) and the previously unreported 10-bromo-1,7-dien-3-ol (**7**) (Figure 2). Sesquiterpenes **1** to **6** were identified in comparison with their spectroscopic data against literature data [21]-[23].

Sesquiterpenes **1** to **6** have been found in other *Laurencia* algae: *L. okumurai*, (**1**, **2, 3, 4,** and **6**) [21],[22], *L. gracilis* (**2**) [24],[25], *L. tristicha* (**5**) [19], and *L. distichophylla* (**3**) [26]. This work represents the first identification of these sequiterpenes in *L. pacifica*.

Sesquiterpene **7** was isolated in very small quantities (approximately 100 ng) from 2 kg of algae and was identified through a combination of high resolution mass spectrometry, infrared spectroscopy, and heteronuclear multiple bond correlation (HMBC), heteronuclear single quantum correlation (HSQC), correlated spectroscopy (COSY) NMR (see Additional file 1). The spectral data obtained closely matched reported spectral data of the related 10-bromo-7,8-expoxychamigr-1-en-3-ol (**8**) (Figure 2) [26], in which the C6 stereochemistry was determined by detailed NMR analysis and was consistent with our data [27]-[29]. The peak assignment and spectral comparison of **7** and **8** are shown in Table 1. There are three related structures with the C1-C2 double bond, one of which is supported by crystal structure data and is of the same absolute configuration as shown for **7** and **8**. While these data are wholly consistent with our assignment, Suescun et al. have identified another compound with the opposite C6 configuration [29]. We thus consider our absolute configuration assignment as tentative. Notwithstanding this, the spectroscopic data is consistent with the assigned structure and represents a new sesquiterpene from *L. pacifica* (Figure 2).

**Table 1:** **1D and 2D NMR spectroscopic data (and assignments) obtained from sesquiterpene 7 and 1D NMR data for 10-bromo-7,8-expoxychamigr-1-en-3-ol (8)**[26]

		
**Position**	**?**_**C**_**,**^**a**^**type**^**b**^	**?**_**H**_**(H, multi,** ***J*** **Hz)**	^**1**^**H-**^**1**^**H COSY**	**HMBC**	**?**_**C**_^**c**^	**?**_**H**_**(multi,** ***J*** **Hz)**
1	136.4, CH	5.84 (1H, d, 10.4)	2	6	136.1	5.84 (dd, 10.4, 1.0)
2	131.1, CH	5.53 (1H, d, 10.4)	1	3, 6, 15	131.3	5.73 (dd, 10.4, 1.6)
3	67.5, *q*C	-	-	-	66.8	-
4	36.1, CH_2_	1.80 (1H, m), 1.73 (1H, m)	5	3	35.0	1.79 (m), 1.81 (m)
5	28.2, CH_2_	1.98 (2H, m)	4	3, 6	23.1	1.69 (ddd, 13.2, 11.2, 4.7), 1.94 (d, 13.2)
6	48.0, *q*C	-	-	-	45.9	-
7	139.0, *q*C	-	-	-	60.5	-
8	120.2, CH	5.22 (1H, s)	9, 14	-	61.4	2.97 (d, 3.0)
9	35.8, CH_2_	2.78 (1H, m), 2.56 (1H, m)	8, 10	-	35.1	2.40 (ddd, 15.4, 11.4, 3.0), 2.69 (dd, 15.4, 5.8)
10	62.0, CH	4.63 (1H, dd, 10.8, 6.3)	9b	12	58.4	4.28 (dd, 11.4, 5.8)
11	41.5, *q*C	-	-	-	40.0	-
12	17.8, CH_3_	1.01 (3H, s)	13	13	18.2	1.08 (s)
13	26.1, CH_3_	1.10 (3H, s)	12	6, 10, 12	25.8	0.98 (s)
14	21.7, CH_3_	1.55 (3H, s)	9		26.0	1.17 (s)
15	28.6, CH_3_	1.29 (3H, bs)	-		29.3	1.33 (s)

^a^Data recorded in CDCl_3_ calibrated at ?H 7.24 ppm an for ?c 77.0 for residual solvent; ^b^in the case of a diasterotopic pair of hydrogens, `a¿ denotes the downfield proton while `b¿ denotes the up field proton; ^c^chemical shift for the carbons was inferred from HSQC and HMBC data.

Sequiterpenes **1** to **5** were isolated in sufficient quantities allowing direct evaluation as pure compounds against a panel of cancer and non-cancer-derived cell lines. Due to the low levels of sesquiterpenes **6** and **7**, these were screened as a 1:1 mixture (both were isolated from the same extract fraction as determined by ^1^H NMR) [30]. Initial cytotoxicity screening was conducted at a single dose of 25 ?M, and these data are presented in Table 2.

**Table 2:** Percentage growth inhibition by sesquiterpenes (1 to 7)

	**Compound**					
**Cell line**	**1**	**2**	**3**	**4**	**5**	**6/7**
HT29^a^	60?±?3	12?±?2	12?±?5	>100	<10	10?±?5
U87^b^	25?±?2	13?±?6	15?±?7	52?±?2	12?±?4	15?±?6
MCF-7^c^	81?±?6	<10	<10	>100	<10	<10
A2780^d^	65?±?5	<10	<10	99?±?1	<10	<10
H460^e^	11?±?1	<10	<10	71?±?1	<10	<10
A431^f^	86?±?1	<10	<10	>100	<10	<10
Du145^g^	41?±?1	23?±?6	18?±?3	92?±?2	18?±?2	17?±?3
BE2-C^h^	>100	<10	<10	>100	<10	<10
SJ-G2^b^	40?±?5	<10	10?±?3	>100	12?±?3	14?±?3
MIA^i^	47?±?2	12?±?4	17?±?3	92?±?2	18?±?3	19?±?2
SMA^j^	34?±?8	<10	<10	95?±?1	<10	<10
MCF10A^k^	10?±?6	<10	<10	31?±?10	<10	<10

Isoaplysin (**1**), isolaurenisol (**2**), debromoisolaurinterol (**3**), debromoaplysinol (**4**), laur-11-en-10-ol (**5**), and a 1:1 mixture of the 3?-hydroxydebromoaplysin (**6**) and 10-bromo-1,7-dien-3-ol (**7**) against a panel of cancer and non-cancer derived cell lines at a single dose (25 ?M). Values are measured relative to an untreated control. ^a^Colon; ^b^glioblastoma; ^c^breast; ^d^ovarian; ^e^lung; ^f^skin; ^g^prostate; ^h^neuroblastoma; ^i^pancreas; ^j^glioblastoma (murine); ^k^breast (normal).

Analysis of the cytotoxicity data presented in Table 2 highlights the low level of cell death of the 12 cell lines examined on treatment with **2**, **3**, **5,** and **6**/**7**. Isoaplysin (**1**) and debromoaplysinol (**4**) displayed promising levels of cell death from 10% to >100% and 31% to >100% at 25 ?M drug concentration, respectively. Of the other analogues, only isolaurenisol (**2**) displayed any growth inhibition at >20% (Du145, prostate cancer cell line). Given the activities of these three analogues, a full dose response evaluation was undertaken across our panel of carcinoma and normal cell lines [20]. These data are presented in Table 3.

**Table 3:** **Growth inhibition (GI**_**50**_**?M) of isoaplysin (1), isolaurenisol (2), and debromoaplysinol (4) against a panel of cancer and non-cancer derived cell lines**

	**Compound**		
**Cell line**	**1**	**2**	**4**
HT29^a^	15?±?1.2	>50	9.1?±?1.1
U87^b^	40?±?0.6	>50	26?±?1.7
MCF-7^c^	20?±?1.3	>50	14?±?1.7
A2780^d^	17?±?0.6	>50	10?±?1.7
H460^e^	34?±?1.2	>50	18?±?0.3
A431^f^	17?±?0.6	>50	9.6?±?0.9
Du145^g^	12?±?0.3	>50	6.8?±?0.3
BE2-C^h^	27?±?2.3	>50	13?±?0.9
SJ-G2^b^	29?±?0.7	>50	15?±?0.7
MIA^i^	23?±?1.5	>50	16?±?0.7
SMA^j^	24?±?3.8	>50	14?±?1.2
MCF10A^k^	46?±?3.2	>50	28?±?1.0

GI_50_ is the concentration of drug that reduces cell growth by 50%. ^a^Colon; ^b^glioblastoma; ^c^breast; ^d^ovarian; ^e^lung; ^f^skin; ^g^prostate; ^h^neuroblastoma; ^i^pancreas; ^j^glioblastoma (murine); ^k^breast (normal).

As anticipated, isolaurenisol (**2**) displayed no noteworthy cytotoxicity returning a GI_50_ value?>?50 ?M across all cell lines examined. Both isoaplysin (**1**) and debromoaplysinol (**4**) displayed good to excellent levels of cytotoxicity. Isoaplysin (**1**) returned an average GI_50_ value of 23 ?M (from 15?±?1.2 ?M to 40?±?0.6 ?M against the HT29 and U87 cell lines, respectively), while debromoaplysinol (**4**) returned an average GI_50_ value of 14 ?M (from 6.8?±?0.3 ?M to 26?±?1.7 ?M against the Du145 and U87 cell lines, respectively) when screened in cancer-derived cell lines. Both compounds showed the greatest growth inhibitory effect in the prostate cancer-derived cell line Du145 with GI_50_ values of 12 and 6.8 ?M, respectively, and the least growth inhibitory effect in the non-cancer-derived normal breast cells with GI_50_ values of 46 and 28 ?M, respectively. Indeed, **1** and **4** were up to fourfold more potent in cancer-derived cell populations than normal cells, imbuing them with properties favorable for future development as anti-cancer agents.

Given the structural similarity of the isolated analogues (**1** to **6**), being direct structural homologues or biosynthetically related, the observed differences in cytotoxicity suggest that the presence of the furan moiety and positioning and nature of the pendent substituents was important for cytotoxicity. Analogues **1** and **4** are Br???OH bioisosteres, and **4** and **6** are positional isomers (C3a-OH (**1**) and a C10a-OH (**6**)). The position of the???OH moiety is clearly important with the 3?-hydroxydebromoaplysin (**6**) devoid of cytotoxicity, whereas debromoaplysinol (**4**) displays excellent levels of activity (for a potential lead compound) against the HT29 (9.1 ?M), A431 (9.6 ?M), and Du145 (6.8 ?M) cell lines [31]. Isoaplysin (**1**) and debromoaplysinol (**4**) differ only in the presence of the C3a-Br (**1**) and a C3a-OH (**4**) moiety, and **4** displays enhanced specific and broad spectrum cytotoxicity relative to **1**, suggesting that the???OH moiety enhances the cytotoxicity of this class of compounds.

Sun et al. isolated and screened the related sesquiterpenes: aplysin-9-ene, epiaplysinol, debromoepiaplysinol, aplysinol, and aplysin isolated from *Laurencia tristicha* in a MTT assay against lung adenocarcinoma (A549), stomach cancer (BGC-823), hepatoma (Bel 7402), colon cancer (HCT-8), and HeLa cell lines [19]. Interestingly, only debromoepiaplysinol (epi-**4**, this work) displayed cytotoxicity with a GI_50_?=?15.5 ?M against HeLa cells, where as herein, debromoaplysinol (**4**) displays activity across our panel of 11 cancer cell lines with strong activity in the HT29 (9.1 ?M), A431 (9.6 ?M), and Du145 (6.8 ?M) cells [19]. These findings serve to emphasize the subtle nature of drug-ligand interactions and the role of stereochemistry in eliciting biological activity, c.f. **1**, **4,** and **6**.

### Conclusions

Herein, we have identified sesquiterpenes **1** to **6** for the first time in *L. pacifica* and isolated a new sesquiterpene and 10-bromo-1,7-dien-3-ol (**7**). Screening of these analogues against 11 cancer cell lines revealed modest to good levels of cytotoxicity for **1** and **4**, with up to fourfold selectivity towards cancer-derived cell populations compared with normal cells. Given the low molecular weights and high F*sp*^3^ content, the structure activity data elucidated in this small subset of analogues, we believe that **1** and **4** represent excellent leads for the development of selective and potent anticancer agents [31].

#### Experimental

##### General experimental

*Solvents*. Solvents used for TLC, speedy column, and centrifugal chromatography were of bulk quality and were distilled from glass prior to use. In the case of HPLC, all of the solvents were HPLC grade and were filtered and degassed prior to their use. The solvent referred as LP (light petroleum), is a mixture of different alkanes with a boiling point 60°C to 80°C.

##### Collection of the Mexican algae

*L. pacifica* algae were collected on the coast of the Baja California Peninsula, Mexico. The algae was cleaned of epiphytes, rinsed with fresh water, and dried in the sun at the collection site. The specimens were stored at ?20°C. A voucher specimen of *L. pacifica* was preserved on location in 5% formaldehyde and deposited in a private collection at the Algal Laboratory in the Interdisciplinary Center of Marine Sciences (CICIMAR), La Paz, B.C.S., Mexico, for taxonomical identification and future reference. Subsequently, in the laboratory, 10 g of dry algae was roughly torn or cut to small pieces and then ground with a mortar and pestle. The powdered algae was then submerged in 250 mL of ethanol. The mixture was left for 48 h at 25°C to 35°C. Afterwards, the mixture was filtered and the residual algal tissue was extracted again under the same conditions. Both filtered extracts were combined and concentrated to dryness under reduced pressure at 40°C to obtain *ca* 30 mg of extract. These extracts were used for biological screening.

##### Extracts of L. pacifica and its fractionation

Crude extract of *L. pacifica* 2 kg of algae was reduced to small pieces of *ca* as before, and then submerged in 1 L of ethanol. The resulting mixture was left for 48 h at 25°C to 35°C. Afterwards, the mixture was filtered and the residual algal tissue was extracted again under the same conditions. Both filtered extracts were combined and concentrated to dryness under reduced pressure at 40°C to obtain 2.2 g of extract. Fractionation of the crude extract was commenced with a speedy column resulting in 18 fractions [32]. All fractions were tested in the colorimetric assay; active fractions were then fractionated in normal phase HPLC until isolation of a pure compound.

##### NMR

Proton and ^13^C NMR spectra were recorded on a Bruker Ascend 400 or Bruker Ascend 600 (Madison, WI, USA). All NMR spectra were recorded as CDCl_3_ solutions; the solvent signal was used as internal standard for chemical shifts (^13^C *?* 77.0 ppm, and ^1^H ? 7.24 ppm for the residual CHCl_3_ proton). All spectra, including HSQC, HMBC, distortionless enhancement by polarization transfer (DEPT135), distortionless enhancement by polarization transfer with retention of quaternaries (DEPTQ135), and (homonuclear) COSY-utilized standard Bruker pulse programs.

##### Cell culture and stock solutions

Stock solutions were prepared as follows and stored at ?20°C: drugs were stored as 20 mM solutions in DMSO. All cell lines were cultured at 37°C, under 5% CO_2_ in air. All cancer-derived cells lines were maintained in Dulbecco's modified Eagle's medium (Trace Biosciences, Sydney, Australia) supplemented with 10% fetal bovine serum, 10 mM sodium bicarbonate, penicillin (100 IU/mL), streptomycin (100 ?g/mL), and glutamine (4 mM). The non-cancer-derived breast cell line MCF10A was maintained in Dulbecco's modified Eagle's medium and Ham's F12 medium (1:1, Trace Biosciences, Sydney, Australia) supplemented with 5% heat inactivated horse serum, HEPES (20 mM), penicillin (100 IU/ml), streptomycin (100 ?g/mL), glutamine (2 mM), epidermal growth factor (20 ng/ml), hydrocortisone (500 mg/ml), cholera toxin (100 ng/ml), and insulin (10 ?g/mL).

##### In vitro growth inhibition assay

Cells in logarithmic growth were transferred to 96-well plates. Cytotoxicity was determined by plating cells in duplicate in 100 mL medium at a density of 2,500 to 4,000 cells/well. On day 0, (24 h after plating) when the cells were in logarithmic growth, 100 ?L medium with or without the test agent was added to each well. After 72 h, drug exposure growth inhibitory effects were evaluated using the MTT (3-[4,5-dimethyltiazol-2-yl]-2,5-diphenyl-tetrazolium bromide) assay and absorbance read at 540 nm. Percentage growth inhibition was determined at a fixed drug concentration of 25 ?M. A value of 100% is indicative of total cell growth inhibition. Those analogues showing appreciable percentage growth inhibition underwent further dose response analysis allowing for the calculation of a GI_50_ value. This value is the drug concentration at which cell growth is 50% inhibited based on the difference between the optical density values on day 0 and those at the end of drug exposure [20].

*Isoaplysin: (3S,3aS,8bS)-3a-(Bromomethyl)-3,6,8b-trimethyl-2,3,3a,8b-tetrahydro-1H-benzo[b]cyclopenta[d]furan (****1****)*

Isolated as a white powder: 1.5 mg; [?] ${}_{D}^{20}$ = ?5.3° (*c* 0.001, CH_3_OH); IR?_max_ 2,927 (C¿H), 2,858, 1,620 (C?=?C), 1,593 (C?=?C), 1,499, 1,453 (C?=?C), 1,423, 1,376 (C¿H), 1,268 (C¿O), 1,135, 946 cm^?1^; ^1^H NMR (400 MHz, CDCl_3_) *?* 6.88 (d, *J*?=?7.6 Hz, 1H, H-5), 6.66 (dd, *J*?=?7.5, 0.7 Hz, 1H, H-4), 6.58 (s, 1H, H-2), 3.66, 3.56 (ABq, *J*_AB_?=?11.1 Hz, 2H, H-12), 2.27 (s, 3H, H-15), 2.21 to 2.12 (m, 1H, H-10), 1.90 to 1.83 (m, 1H, H-8a), 1.70 to 1.60 (m, 2H, H-8b, H-9a), 1.50 (s, 3H, H-14), 1.20 to 1.13 (m, 1H, H-9b), 1.10 (d, *J*?=?6.7 Hz, 3H, H-13). ^13^C NMR (101 MHz, CDCl_3_) *?* 158.8 (*q*C, C-1), 138.3 (*q*C, C-3), 133.0 (*q*C, C-6), 122.2 (CH, C-5), 121.4 (CH, C-4), 109.3 (CH, C-2), 97.2 (*q*C, C-11), 55.5 (*q*C, C-7), 43.7 (CH, C-10), 42.6 (CH_2_, C-8), 34.6 (CH_2_, C-12), 31.5 (CH_2_, C-9), 22.9 (CH_3_, C-14), 21.5 (CH_3_, C-15), 13.8 (CH_3_, C-13).

*Isolaurenisol: 2-[3-(Bromomethylene)-1,2-dimethylcyclopentyl]-5-methylphenol (****2****)*

Isolated as a white solid: 2.5 mg; [?] ${}_{D}^{20}$ = ?7.2° (*c* 0.0025, CH_3_OH); IR?_max_ 3,513 (O-H), 2,959 (C-H), 2,929, 2,870, 1,616 (C?=?C), 1,576 (C?=?C), 1,514, 1,454, 1,412, 1,294 (C-H), 1,254 (C-O), 1,186, 1,123, 809, 787, 653 (C-Br) cm^?1^; ^1^H NMR (400 MHz, CDCl_3_) *?* 7.15 (d, *J*?=?7.9 Hz, 1H, H-5), 6.68 (dd, *J*?=?7.9, 1.0 Hz, 1H, H-4), 6.57 (d, *J*?=?1.1 Hz, 1H, H-2), 5.99 (d, *J*?=?2.0 Hz, 1H, H-13), 5.06 (s, 1H, OH), 3.05 to 2.95 (m, 1H, H-11), 2.56 (dt, *J*?=?12.9, 7.2 Hz, 1H, H-8a), 2.25 (s, 3H, H-15), 2.07 to 1.96 (m, 1H, H-9a), 1.64 to 1.57 (m, 1H, H-8b), 1.45 (s, 3H, H-14), 1.42 to 1.36 (m, 1H, H-9b), 1.22 (d, *J*?=?7.2 Hz, 3H, H-12). ^13^C NMR (101 MHz, CDCl_3_) *?* 160.2 (*q*C, C-10), 153.3 (*q*C, C-1), 138.0 (*q*C, C-3), 128.7 (*q*C, C-6), 128.1 (CH, C-5), 121.3 (CH, C-4), 118.2 (CH, C-2), 101.3 (CH, C-13), 52.0 (*q*C, C-7), 39.2 (CH, C-11), 39.2 (CH_2_, C-8), 31.0 (CH_2_, C-9), 26.8 (CH_3_, H-14), 20.7 (CH_3_, C-15), 19.1 (CH_3_, C-12).

*Debromoisolaurinterol: [(1* ***R****,3* ***S****)-1,3-dimethyl-2-methylenecyclopentyl]-5-methyl-2-phenol (****3****)*

Isolated as a colorless oil: *ca* 1 mg; IR?_max_ 3,458 (O-H), 3,055 (C-H), 2,984 (C-H), 1,641 (C?=?C), 1,581 (C?=?C), 1,113 (C-H) cm^?1^; ^1^H NMR (400 MHz, CDCl_3_) *?* 7.21 (d, *J*?=?8.0 Hz, 1H, H-5), 6.71 (dd, *J*?=?8.0, 1.1 Hz, 1H, H-4), 6.65 (d, *J*?=?1.4 Hz, 1H, H-2), 5.54 (s, 1H, OH), 5.08 (d, *J*?=?2.1 Hz, 1H, H-12a), 4.93 (d, *J*?=?2.4 Hz, 1H, H-12b), 2.88 to 2.78 (m, 1H, H-10), 2.26 (s, 3H, H-15), 2.25 to 2.18 (m, 1H, H-8a), 2.07 to 1.98 (m, 1H, H-9a), 1.60 to 1.55 (m, 1H, H-8b, obscured), 1.46 (s, 3H, H-14), 1.41 to 1.36 (m, 1H, H-9b), 1.19 (d, *J*?=?7.0 Hz, 3H, H-13).

*Debromoaplysinol: 3a-methanol, 1,2,3,8b-Tetrahydro-3,6,8b-trimethylcyclopenta-3* ***H****-[****b****]benzofuran (****4****)*

Isolated as a colorless oil: *ca* 1 mg; [?] ${}_{D}^{20}$ = 0° (*c* 0.001, CH_3_OH); IR?_max_ 3,425 (O-H), 2,940, 2,871, 1,588 (C?=?C), 1,499, 1,044 (C-O), 860 cm^?1^; ^1^H NMR (400 MHz, CDCl_3_) *?* 6.90 (d, *J*?=?7.6 Hz, 1H, H-5), 6.66 (dd, *J*?=?7.5, 0.7 Hz, 1H, H-4), 6.57 (s, 1H, H-2), 3.84 (dABq, *J*?=?12.4_(AB)_, 4.3 Hz, 1H, H-12a), 3.71 (dABq, *J*?=?12.4_(AB)_, 8.6 Hz, 1H, H-12b), 2.28 (s, 3H, H-15), 1.88 to 1.78 (m, 2H, H-10, H-8a), 1.72 (dd, *J*?=?8.6, 4.3 Hz, 1H, OH), 1.66 to 1.60 (m, 2H, H-8b, H-9a), 1.46 (s, 3H, H-14), 1.16 to 1.11 (m, 1H, H-9b), 1.08 (d, *J*?=?6.8 Hz, 3H, H-13). ^13^C NMR (101 MHz, CDCl_3_) *?* 159.1 (*q*C, C-1), 138.3 (*q*C, C-3), 133.3 (*q*C, C-6), 122.2 (CH, C-5), 121.2 (CH, C-4), 109.0 (CH, C-2), 99.6 (*q*C, C-11), 63.9 (CH_2_, C-12), 54.2 (*q*C, C-7), 42.3 (CH, C-10), 42.3 (CH_2_, C-8), 31.5 (CH_2_, C-9), 22.9 (CH_3_, C-14), 21.2 (CH_3_, C-51), 13.7 (CH_3_, C-13).

*Laur-11-en-10-ol. 3-(4?-Methylphenyl)-1,3,dimethyl-2-methylidenecyclopentanol (****5****)*

Isolated as a colorless oil: *ca* 1 mg; ^1^H NMR (600 MHz, CDCl_3_) *?* 7.21 (d, *J*?=?7.9 Hz, 2H, H-1, H-5), 7.07 (d, *J*?=?8.1 Hz, 2H, H-2, H-4), 5.44 (s, 1H, H-12a), 4.99 (s, 1H, H-12b), 3.47 (d, *J*?=?5.9 Hz, 1H, OH), 2.29 (s, 3H, H-15), 2.08 to 2.04 (m, 1H, H-8a), 1.98 to 1.92 (m, 1H, H-8b), 1.82 to 1.77 (m, 1H, H-9a), 1.67 to 1.62 (m, 1H, H-9b, obscured), 1.48 (s, 3H, H-14), 1.36 (s, 3H, H-13). ^13^C NMR (151 MHz, CDCl_3_) ? 166.4 (*q*C, C-11), 145.4 (*q*C, C-6), 135.3 (*q*C, C-3), 129.0 (2?×?CH, C-2, C-4), 126.3 (2?×?CH, C-1, C-5), 108.6 (CH_2_, C-12), 79.3 (*q*C, C-10), 50.3 (*q*C, C-7), 39.5 (CH_2_, C-8), 39.2 (CH_2_, C-9), 30.8 (CH_3_, C-14), 28.4 (CH_3_, C-13), 21.2 (CH_3_, C-15).

*10?-Hydroxyldebromoepiaplysin. (?)-2,3,3a,8b-Tetrahydro-3-hydroxy-3,3a,6,8b-tetramethyl-1H-benzocyclopentafuran (****6****)*

Isolated as a colorless oil: *ca* 1 mg; ^1^H NMR (600 MHz, CDCl_3_) ? 6.92 (d, *J*?=?7.6 Hz, 1H, H-5), 6.66 (d, *J*?=?7.4 Hz, 1H, H-4), 6.49 (s, 1H, H-2), 5.35 (bs, 1H, OH), 2.26 (s, 3H, H-15), 2.01 to 1.96 (m, 1H, H-8a, obscured), 1.76 to 1.71 (m, 1H, H-8b, obscured), 1.61 to 1.57 (m, 2H, H-9, obscured), 1.39 (s, 3H, H-13), 1.38 (s, 3H, H-14), 1.28 (s, 3H, H-12). ^13^C NMR (151 MHz, CDCl_3_) *?* 158.0 (*q*C, C-1), 138.1 (*q*C, C-3), 133.5 (*q*C, C-6), 122.5 (CH, C-5), 121.0 (CH, C-4), 109.6 (CH, C-2), 100.5 (*q*C, C-11), 82.9 (*q*C, C-10), 53.6 (*q*C, C-7), 40.8 (CH_2_, C-8), 37.0 (CH_2_, C-9), 23.4 (CH_3_, C-14), 22.2 (CH_3_, C-13), 21.1 (CH_3_, C-15), 14.8 (CH_3_, C-12).

*10-Bromo-3,7,11,11-tetramethylspiro[5.5]undeca-1,7-dien-3-ol (****7****)*

Isolated as a colorless oil: *ca* 0.1 mg; ^1^H NMR (600 MHz, CDCl_3_) *?* 5.84 (d, *J*?=?10.4 Hz, 1H, H-4), 5.53 (d, *J*?=?10.5 Hz, 1H, H-5), 5.22 (s, 1H, H-8), 4.63 (dd, *J*?=?10.8, 6.3 Hz, 1H, H-10), 2.65 to 2.59 (m, 1H, H-9a), 2.57 to 2.50 (m, 1H, H-9b), 2.02 to 1.96 (m, 2H, H-1), 1.82 to 1.77 (m, 1H, H-2a), 1.76 to 1.71 (m, 1H, H-2b, obscured), 1.55 (s, 3H, H-14, obscured), 1.29 (s, 3H, H-15), 1.10 (s, 3H, H-13), 1.01 (s, 3H, H-12). ^13^C NMR (151 MHz, CDCl_3_) *?* 139.0 (*q*C, C-7), 136.4 (CH, C-4), 131.1 (CH, C-5), 120.7 (CH, C-8), 67.5 (*q*C, C-3), 61.3 (CH, C-10), 47.1 (*q*C, C-6), 41.5 (*q*C, C-11), 36.1 (CH_2_, C-2), 35.8 (CH_2_, C-9), 28.6 (CH_3_, C-15), 28.2 (CH_2_, C-1), 26.1 (CH_3_, C-13), 21.7 (CH_3_, C-14), 17.8 (CH_3_, C-12).

## Competing interests

The authors declare that they have no competing interests.

## Additional file

## Supplementary Material

Additional file 1: Structural characterization. Click here for file
