# Supplementary material for: Cytotoxic compounds from Laurencia pacifica
Source: Org Med Chem Lett. 2014 Sep 20;4:8. doi: 10.1186/s13588-014-0008-8 (PMC4610114; doi:10.1186/s13588-014-0008-8)

# Cytotoxic compounds from *Laurencia pacifica*

Diana A. Zaleta-Pinet,^1^ Ian P. Holland,^1^ Mauricio Muñoz-Ochoa,^2^ J. Ivan Murillo-Alvarez,^2^ Jennette A Sakoff,^3^ Ian A. van Altena,^1^ Adam McCluskey^1*^

^1^ Chemistry, School of Environmental & Life Science, The University of Newcastle, University Drive, Callaghan NSW 2308, Australia. Phone: +61 249216486; Fax: +61 249 215472; Email: [Adam.McCluskey@newcastle.edu.au](mailto:Adam.McCluskey@newcastle.edu.au);

^2^ Development Technology Department, Interdisciplinary Centre of Marine Sciences, National Technological Institute. La Paz, Mexico.

^3^ Department of Medical Oncology, Calvary Mater Newcastle Hospital, Waratah NSW 2298, Australia.

**Supplementary Data**

**Collection of the Mexican algae.**

*Laurencia* *pacifica* algae were collected on the coast of the Baja California Peninsula, Mexico. The algae was cleaned of epiphytes, rinsed with fresh water and dried in the sun at the collection site. The specimen were stored at -20 °C. A voucher specimen of *Laurencia* *pacifica* was preserved on location in 5% formaldehyde and deposited in a private collection at the Algal Laboratory in the Interdisciplinary Center of Marine Sciences (CICIMAR), La Paz, B.C.S., Mexico, for taxonomical identification and future reference. Subsequently, in the laboratory, 10 g of dry algae was roughly torn or cut to ca 2 cm squares and then ground with a mortar and pestle. The powdered algae was then submerged in 250 mL of ethanol. The mixture was left for 48 h at 25-35 °C. Afterwards, the mixture was filtered and the residual algal tissue was extracted again under the same conditions. Both filtered extracts were combined and concentrated to dryness under reduced pressure at 40 °C to obtain *ca* 30 mg of extract. These extracts were used for biological screening.

**Crude extracts of *Laurencia pacifica* and its fractionation.**

Crude extract of *Laurencia pacifica* 2 kg of algae was reduced to pieces of *ca* 2 cm square, as before, and then submerged in 1 L of ethanol. The resulting mixture was left for 48 h at 25-35 °C. Afterwards, the mixture was filtered and the residual algal tissue was extracted again under the same conditions. Both filtered extracts were combined and concentrated to dryness under reduced pressure at 40 °C to obtain 2.2 g of extract. Fractionation of the crude extract was commenced with a speedy column resulting in 18 fractions.^S1^ All fractions were tested in the colorimetric assay, active fractions were then fractionated in normal phase HPLC until isolation of a pure compound.

**General Experimental**

**Solvents**

Solvents used for TLC, speedy column and centrifugal chromatography were of bulk quality and were distilled from glass prior to use. In the case of HPLC, all of the solvents were HPLC grade and were filtered and degassed prior to their use. The solved referred as LP (light petroleum), is a mixture of different alkanes with a boiling point 60-80 °C.

**NMR**

Proton and ^13^C NMR spectra were recorded on a Bruker Ascend 400 or Bruker Ascend 600. All NMR spectra were recorded as CDCl_3_ solutions; the solvent signal was used as internal standard for chemical shifts (^13^C δ 77.0 ppm, and ^1^H δ 7.24 ppm for the residual CHCl_3_ proton). All spectra, including Heteronuclear Single Quantum Correlation (HSQC), Heteronuclear Multiple Bond Correlation (HMBC), Distortionless Enhancement by Polarization Transfer (DEPT135), Distortionless Enhancement by Polarization Transfer with retention of Quaternaries (DEPTQ135), and (homonuclear) Correlated Spectroscopy (COSY) utilized standard Bruker pulse programs.

**Cell culture and stock solutions**

Stock solutions were prepared as follows and stored at -20 ºC: drugs were stored as 20 mM solutions in DMSO. All cell lines were cultured at 37 ºC, under 5% CO_2_ in air. All cancer derived cells lines were maintained in Dulbecco’s modified Eagle’s medium (Trace Biosciences, Australia) supplemented with 10% foetal bovine serum, 10 mM sodium bicarbonate, penicillin (100 IU/mL), streptomycin (100 µg/mL), and glutamine (4 mM). The non-cancer derived breast cell line MCF10A, was maintained in Dulbecco’s modified Eagle’s medium and Ham’s F12 medium (1:1, Trace Biosciences, Australia) supplemented with 5% heat inactivated horse serum, HEPES (20 mM), penicillin (100 IU/ml), streptomycin (100 µg/mL), glutamine (2 mM), epidermal growth factor (20 ng/ml), hydrocortisone (500mg/ml), cholera toxin (100ng/ml) and insulin (10 µg/mL).

**In vitro growth inhibition assay**

Cells in logarithmic growth were transferred to 96-well plates. Cytotoxicity was determined by plating cells in duplicate in 100 mL medium at a density of 2,500-4,000 cells/well. On day 0, (24 h after plating) when the cells were in logarithmic growth, 100 μL medium with or without the test agent was added to each well. After 72 h drug exposure growth inhibitory effects were evaluated using the MTT (3-[4,5-dimethyltiazol-2-yl]-2,5-diphenyl-tetrazolium bromide) assay and absorbance read at 540 nm. Percentage growth inhibition was determined at a fixed drug concentration of 25 μM. A value of 100% is indicative of total cell growth inhibition. Those analogues showing appreciable percentage growth inhibition underwent further dose response analysis allowing for the calculation of a GI_50_ value. This value is the drug concentration at which cell growth is 50% inhibited based on the difference between the optical density values on day 0 and those at the end of drug exposure.^S2^

**References**

S1. Harwood, L. M. *Aldrichimica* *Acta*. **1985**, *18*, 25-26.

S2. Thaqi, A.; Scott, J.L.; Gilbert, J.; Sakoff, J.A.; McCluskey, A. *Eur. J. Med Chem.* 2010, 45, 1717-1723.

**NMR Spectra of 1.**


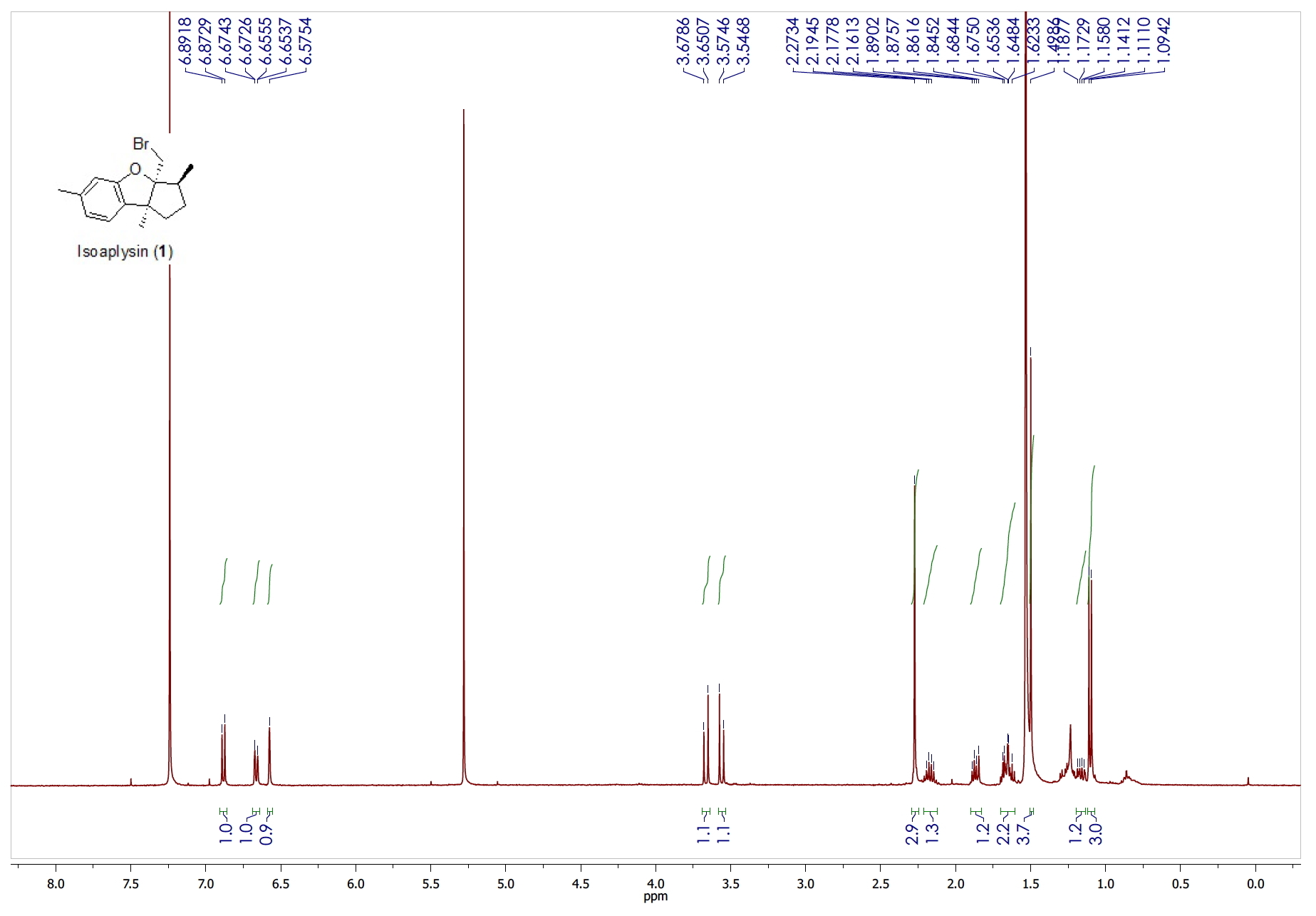


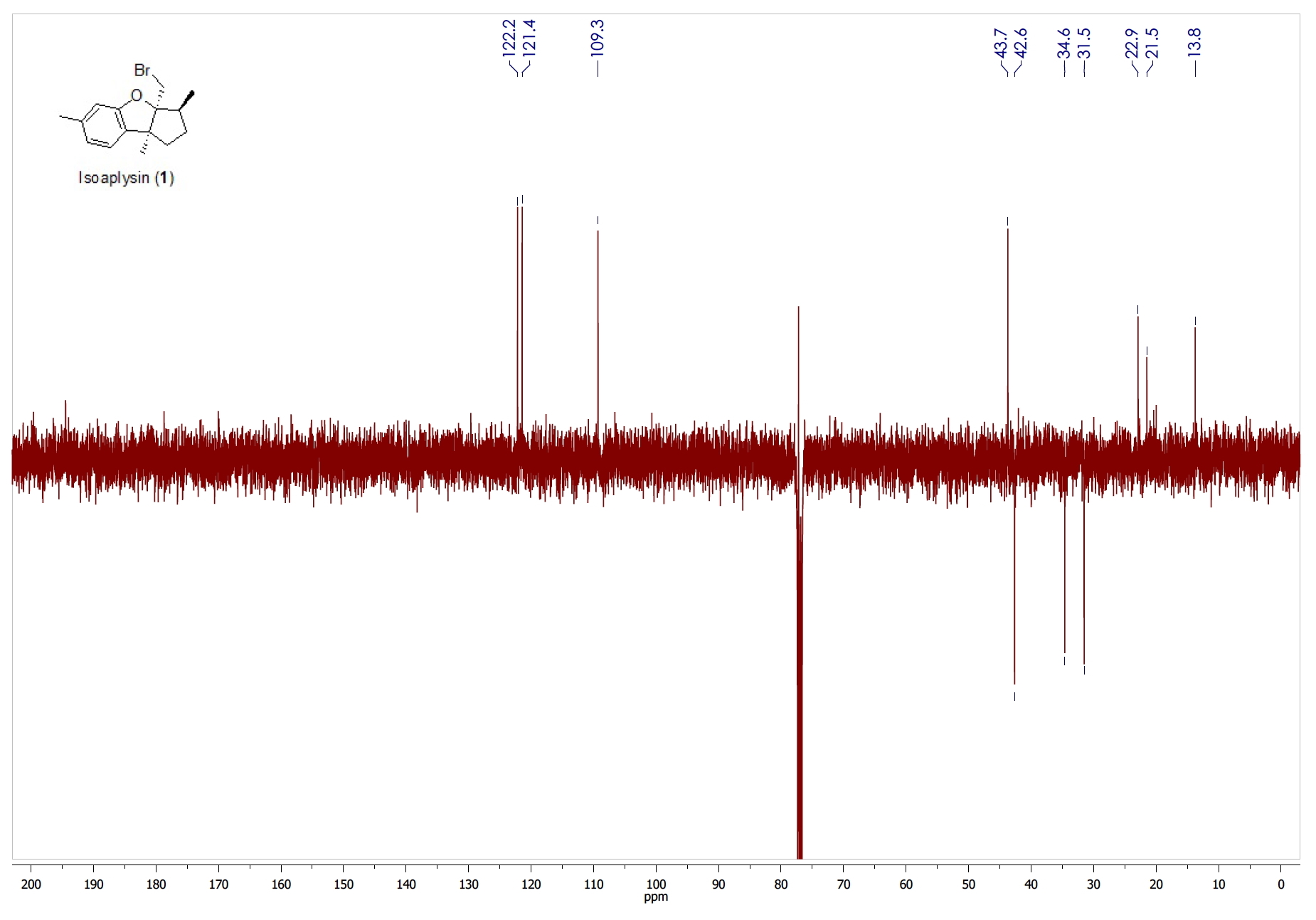


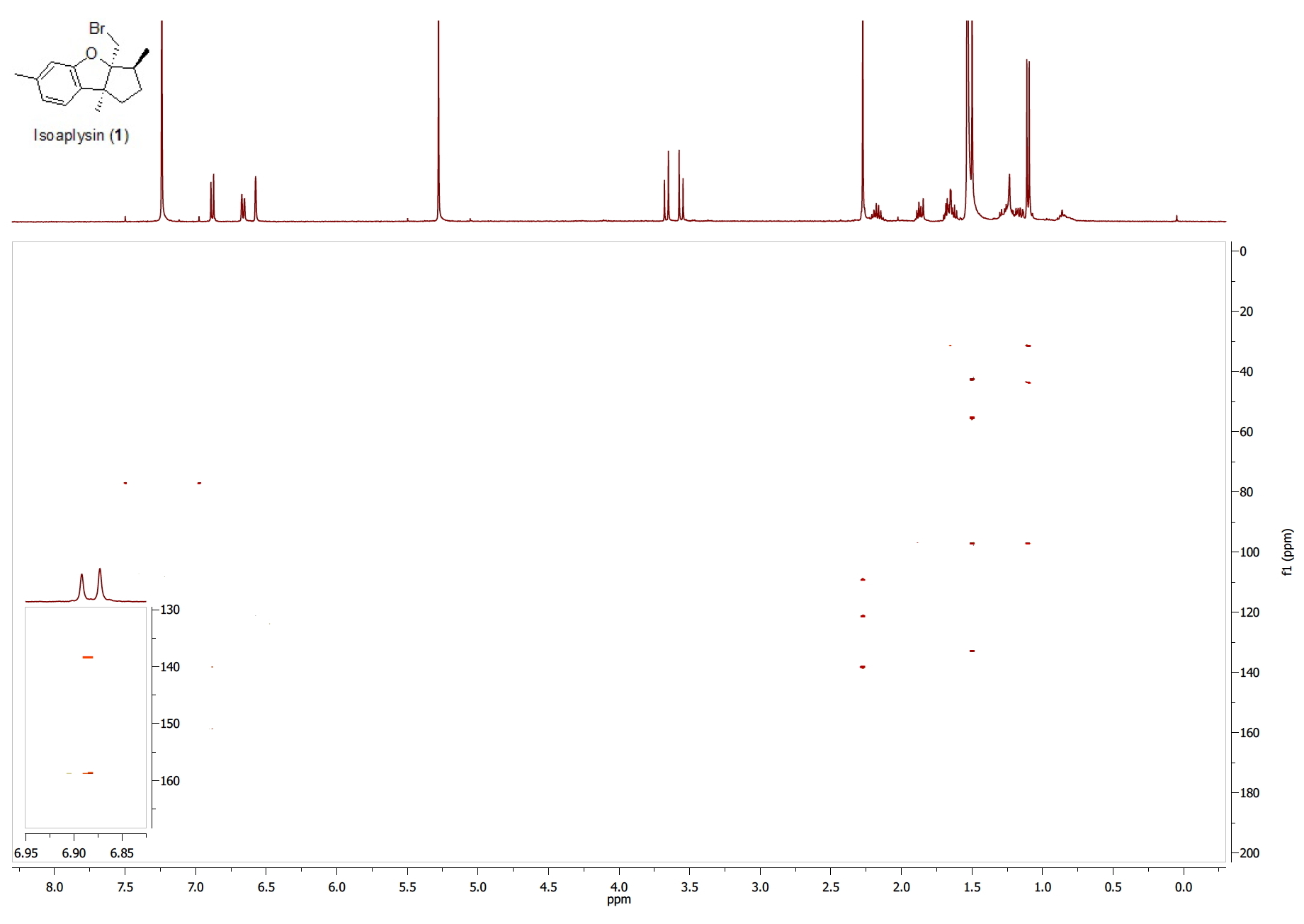


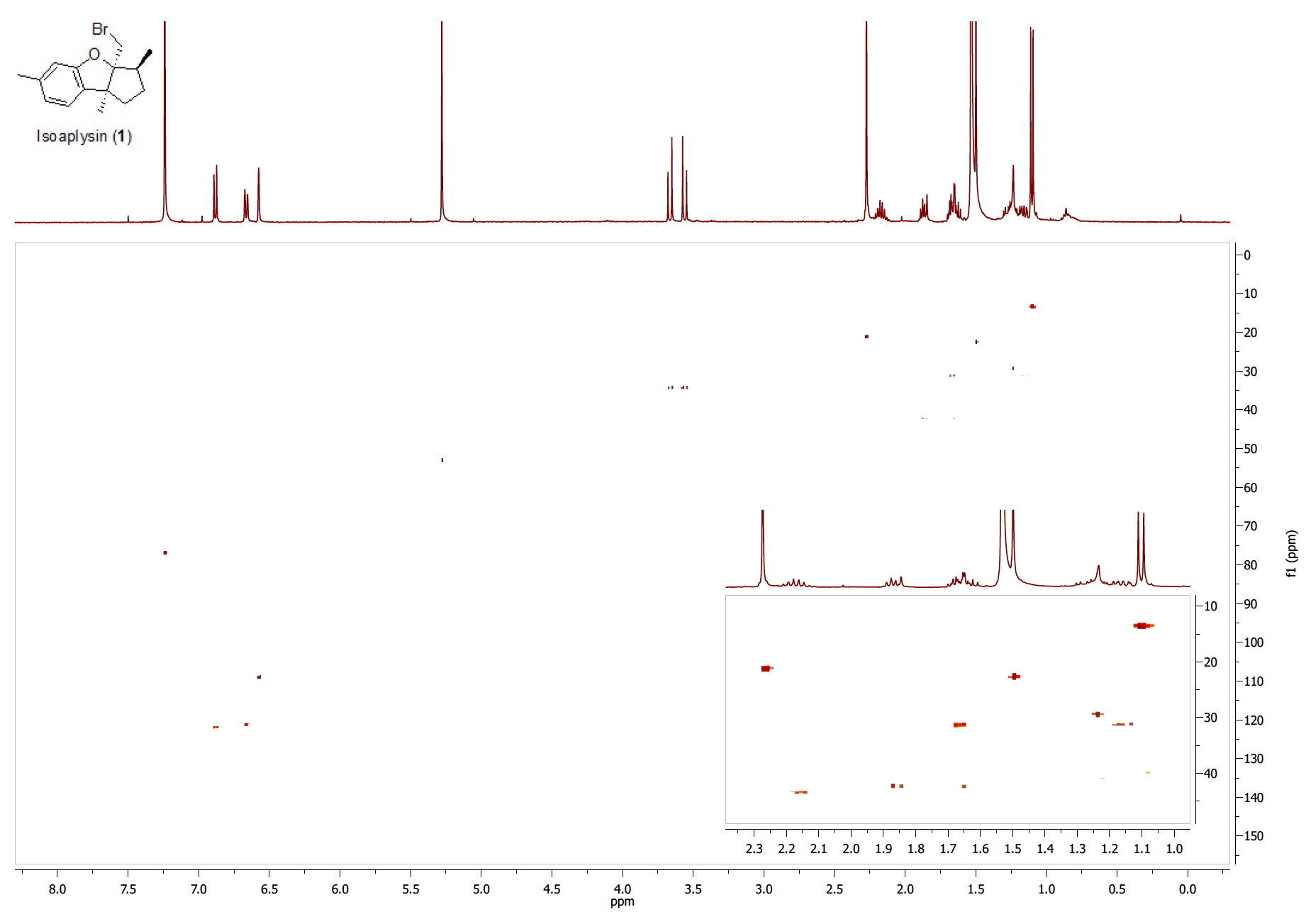


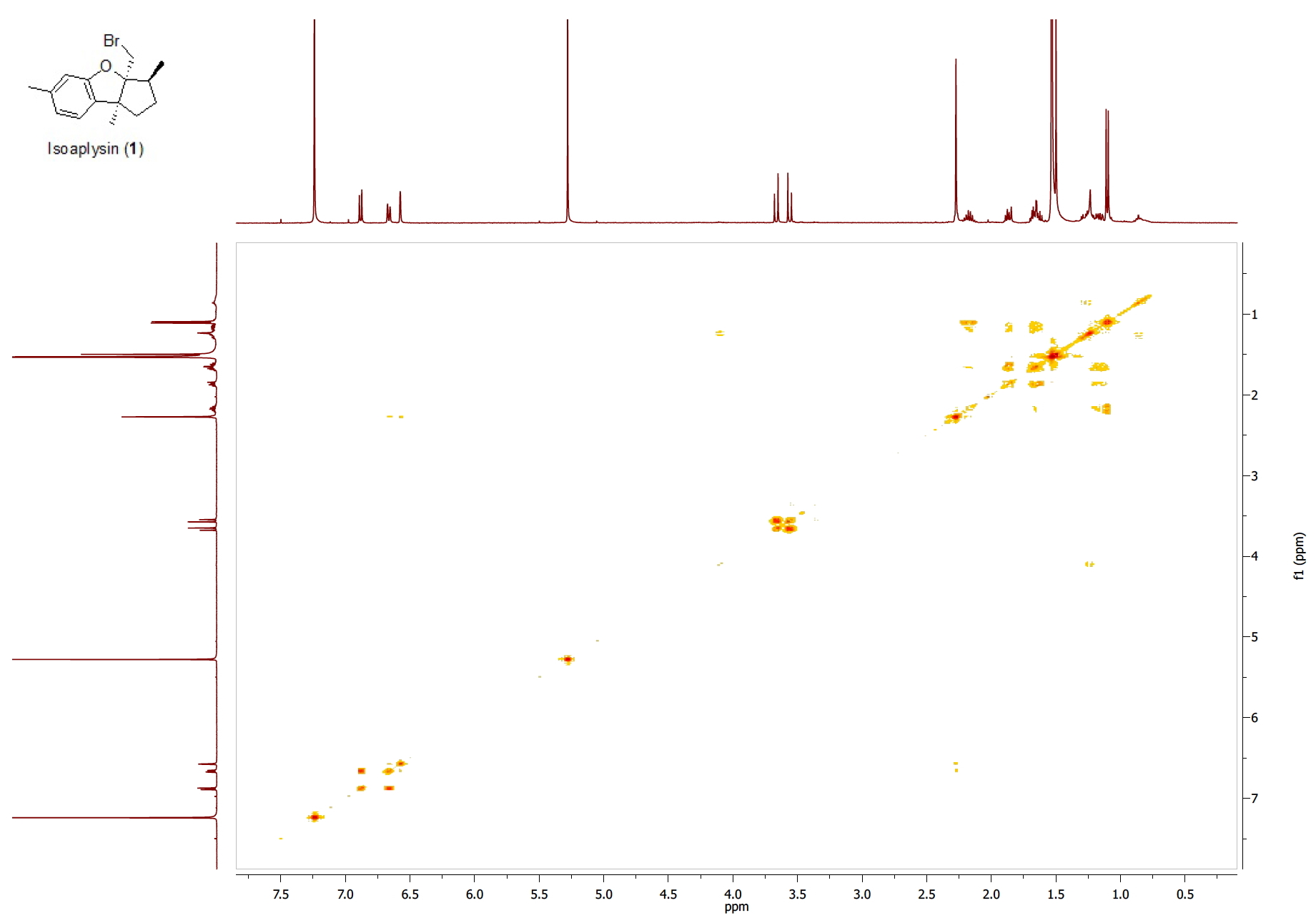


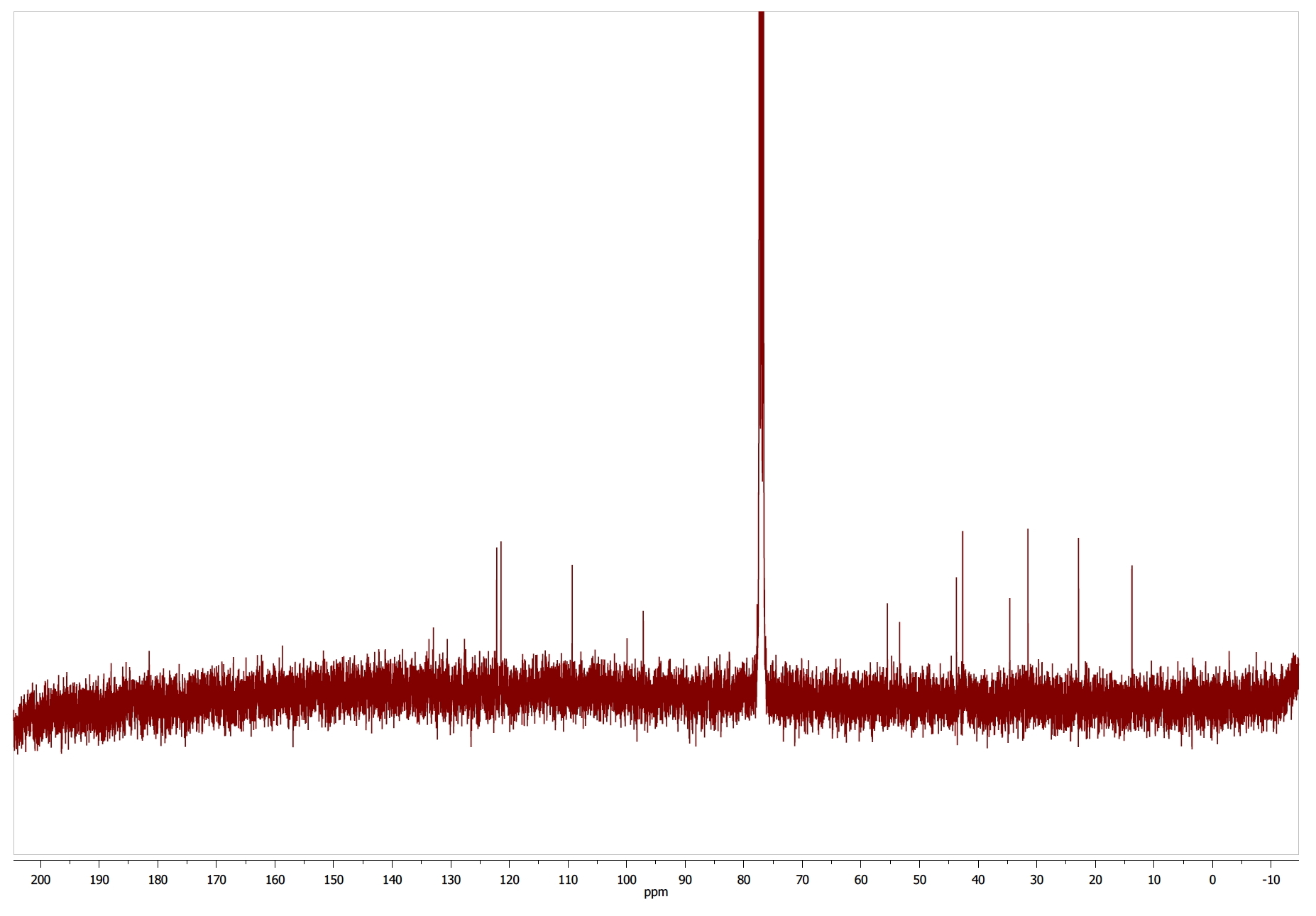


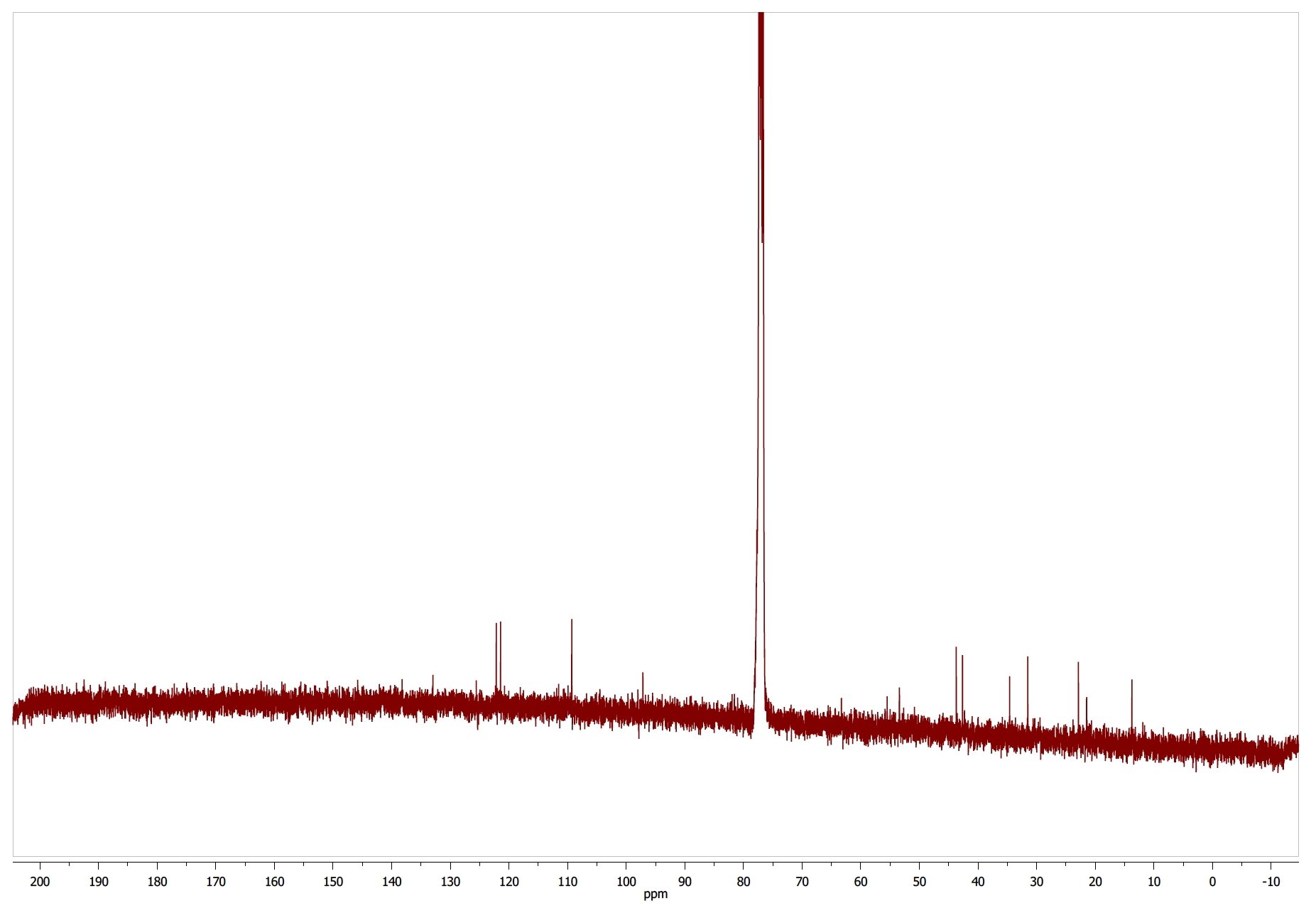


**NMR Spectra of 2.**


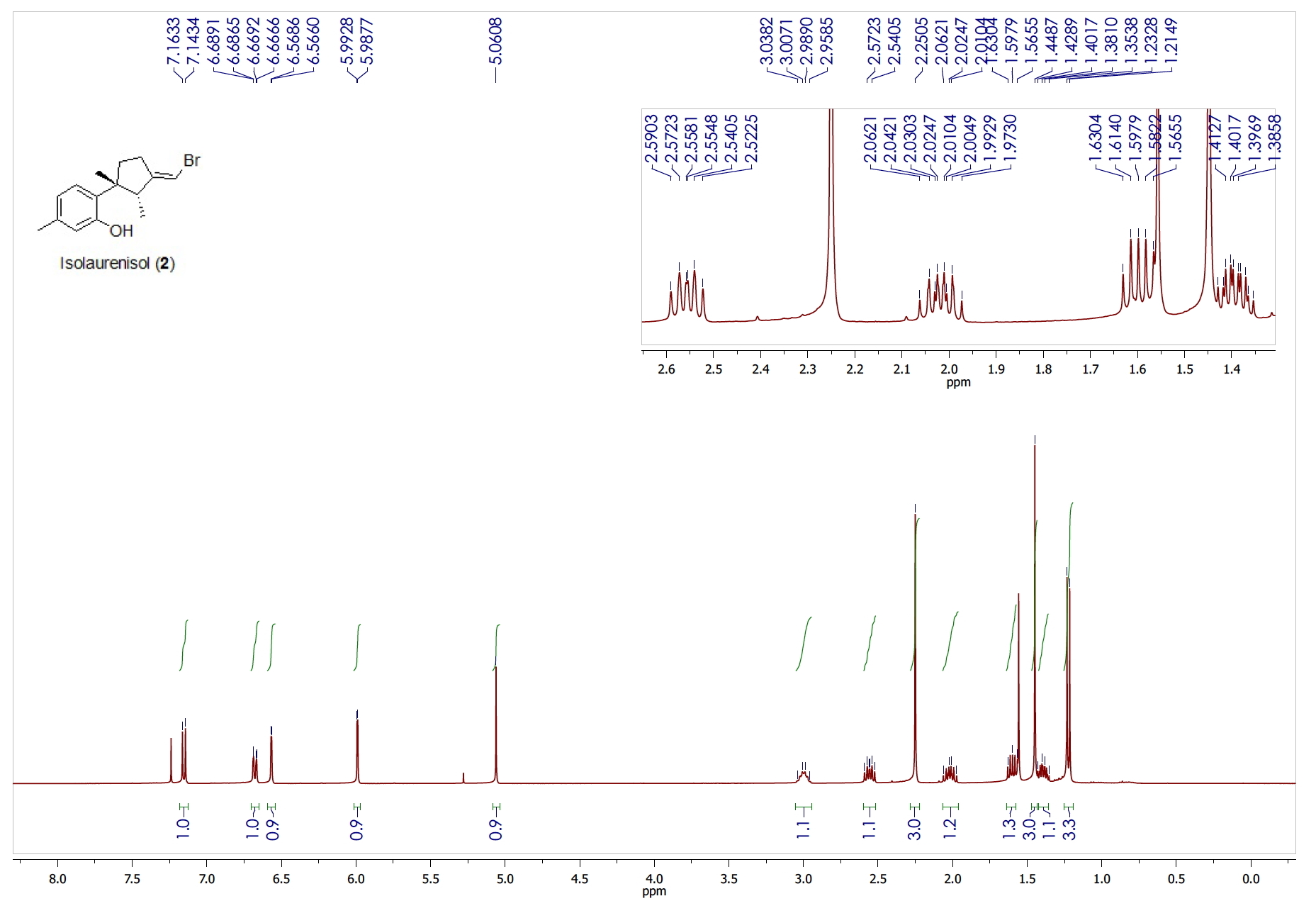


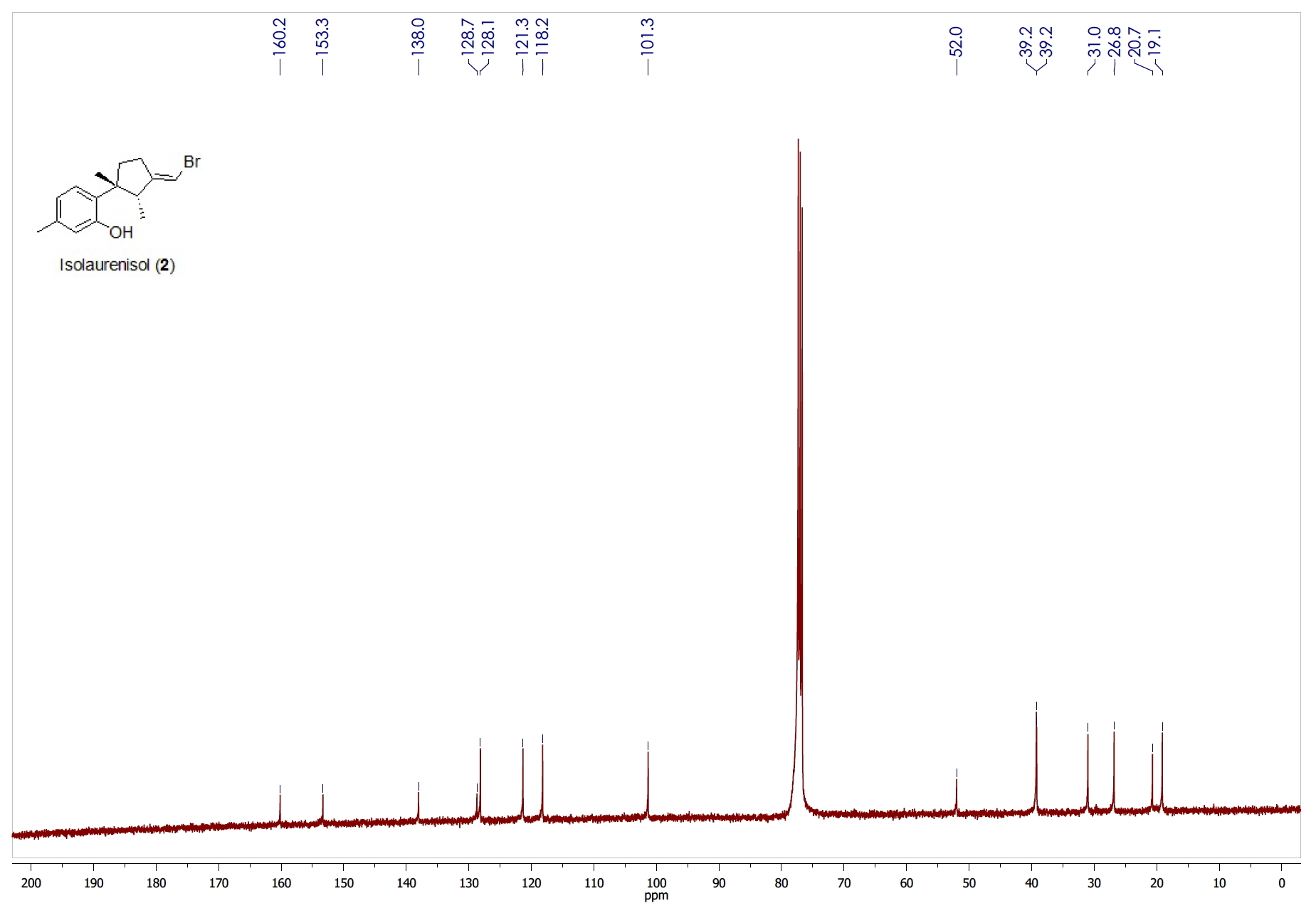


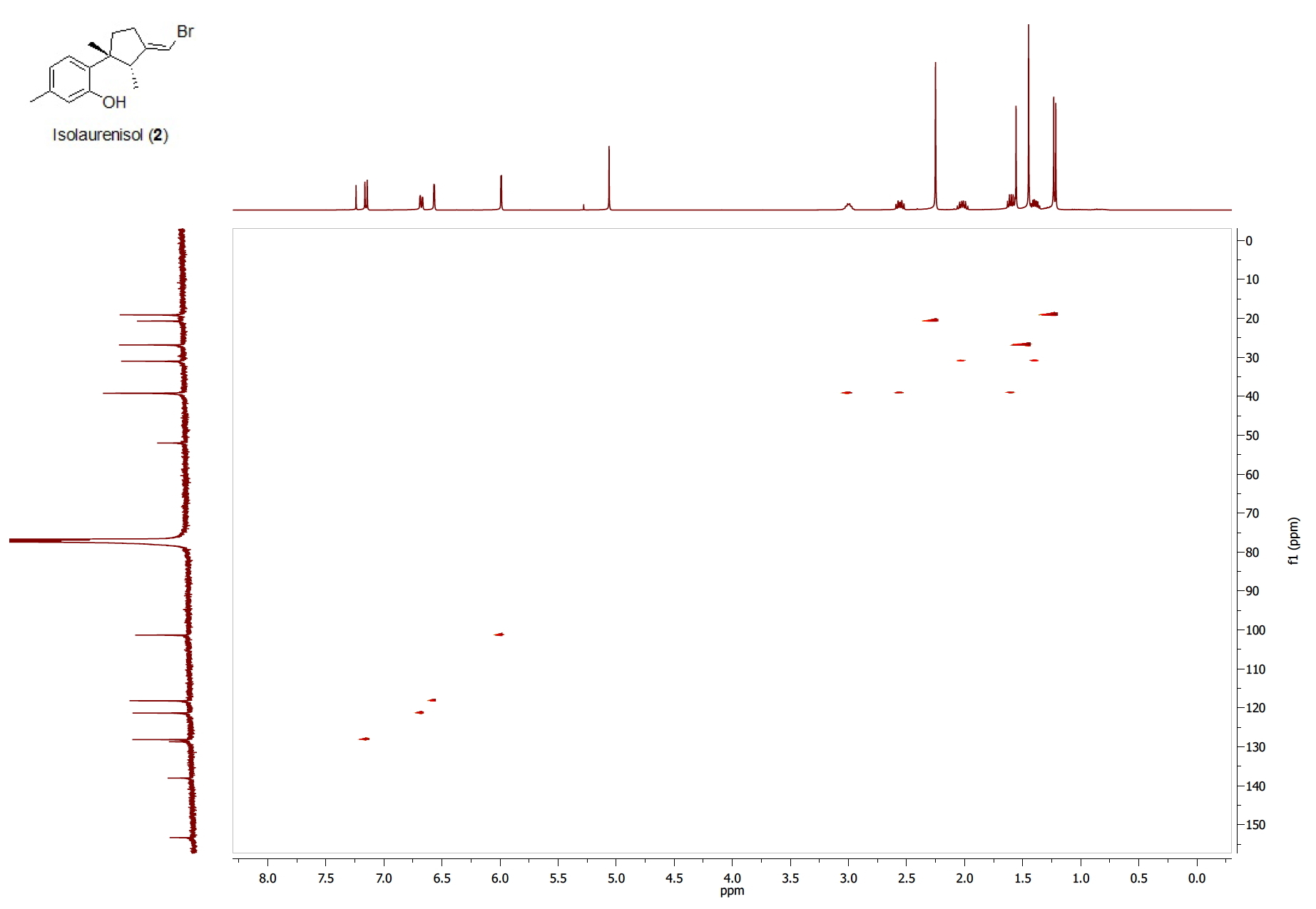


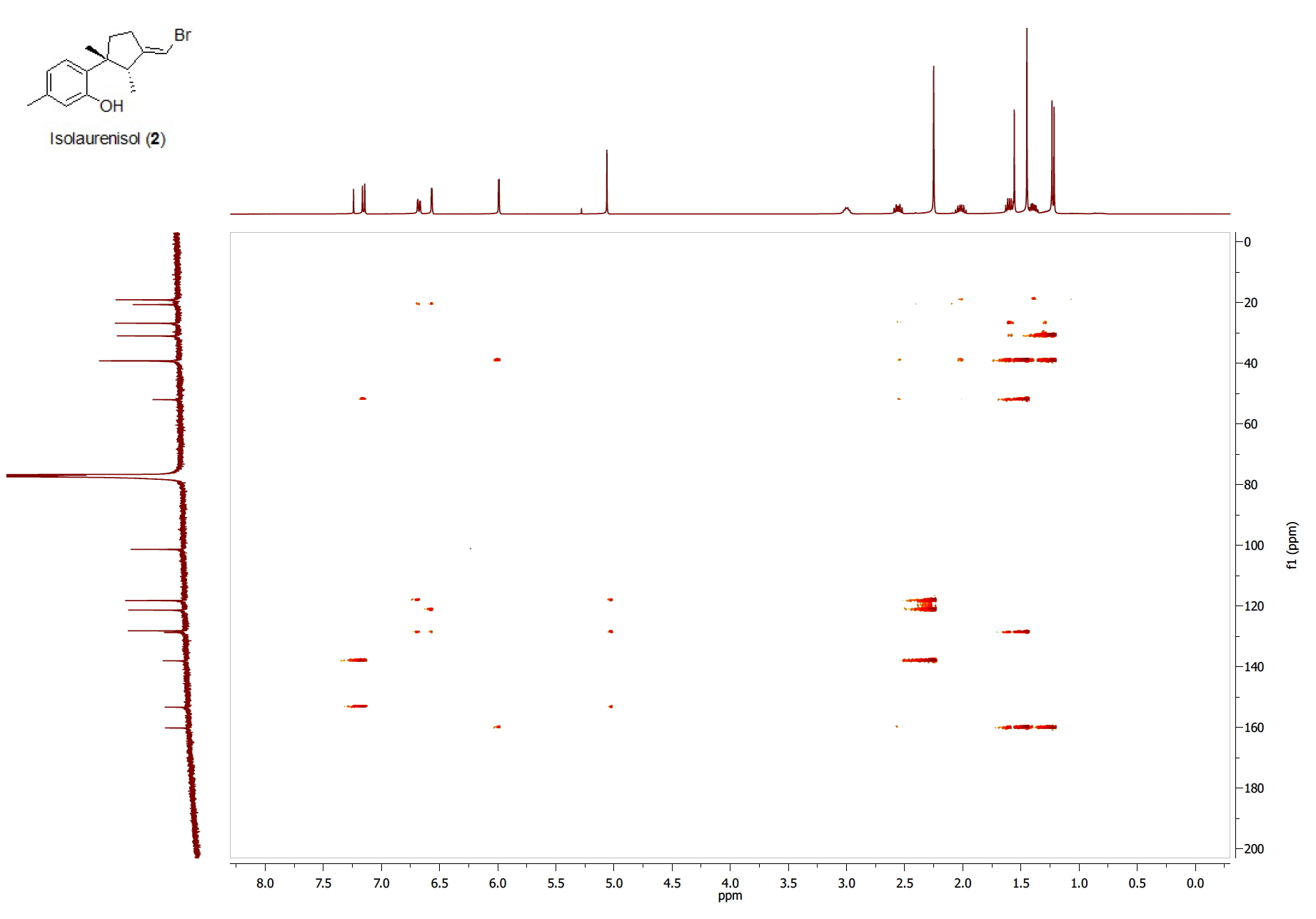


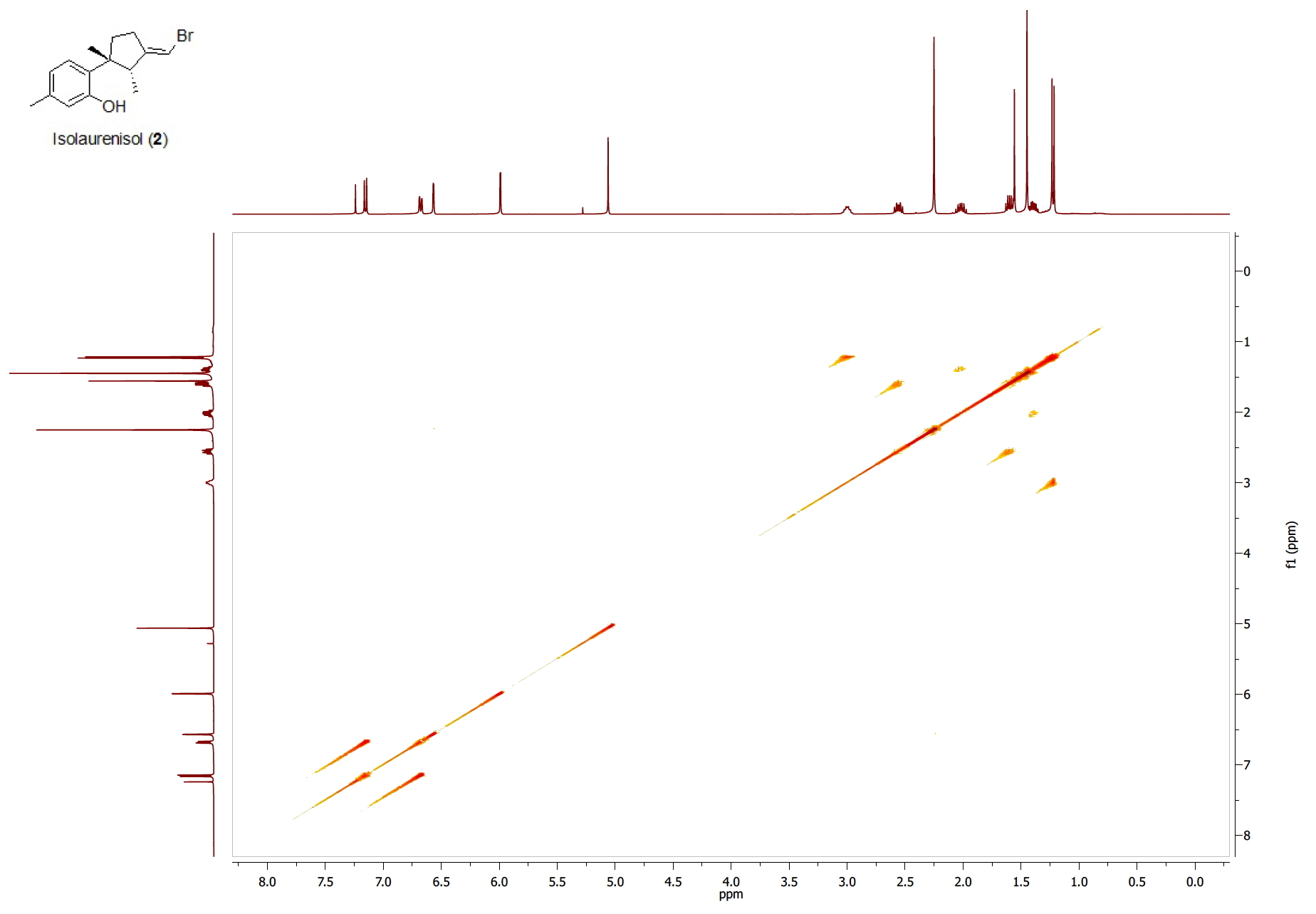


**NMR Spectra of 3.**


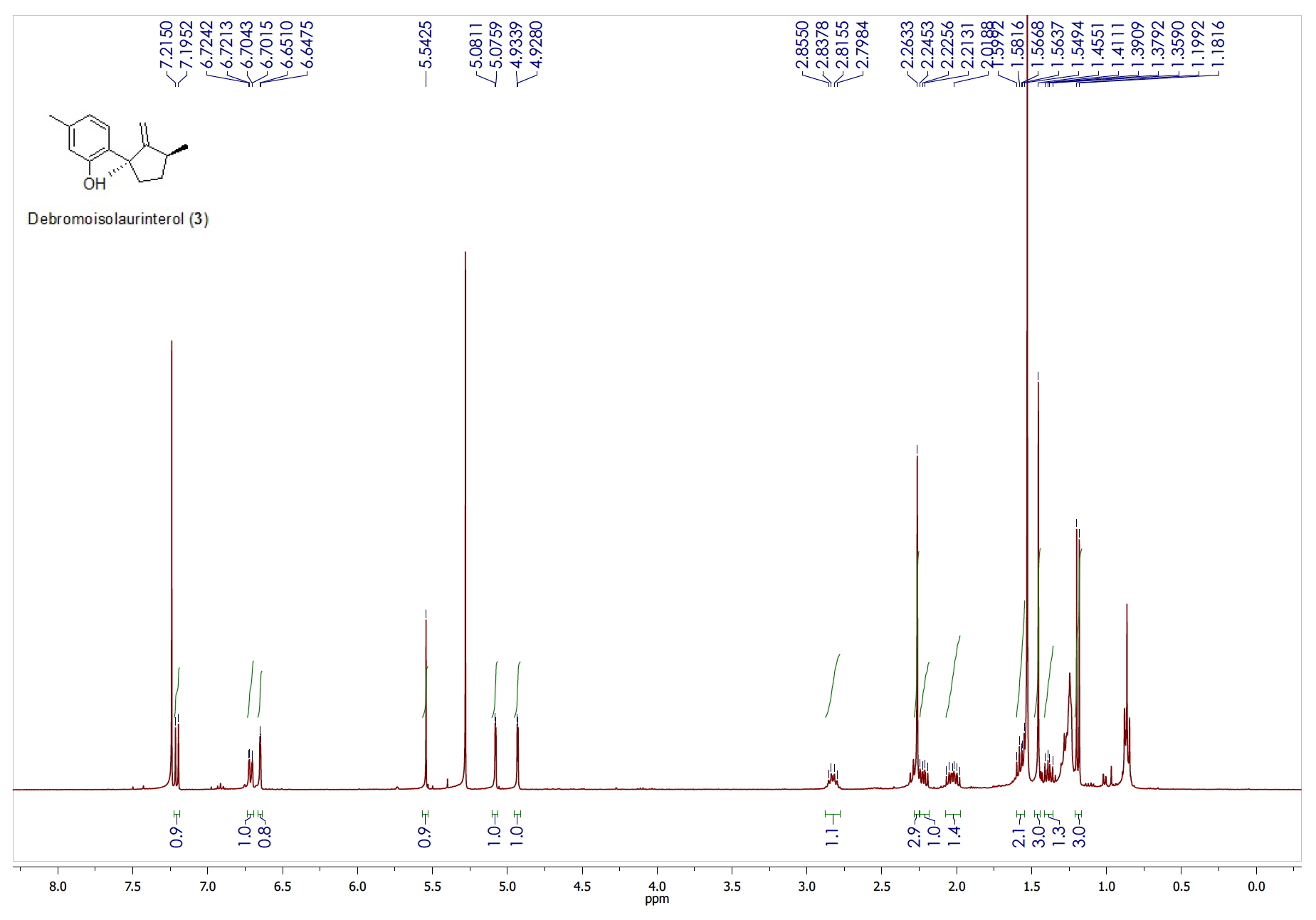


**NMR Spectra of 4.**


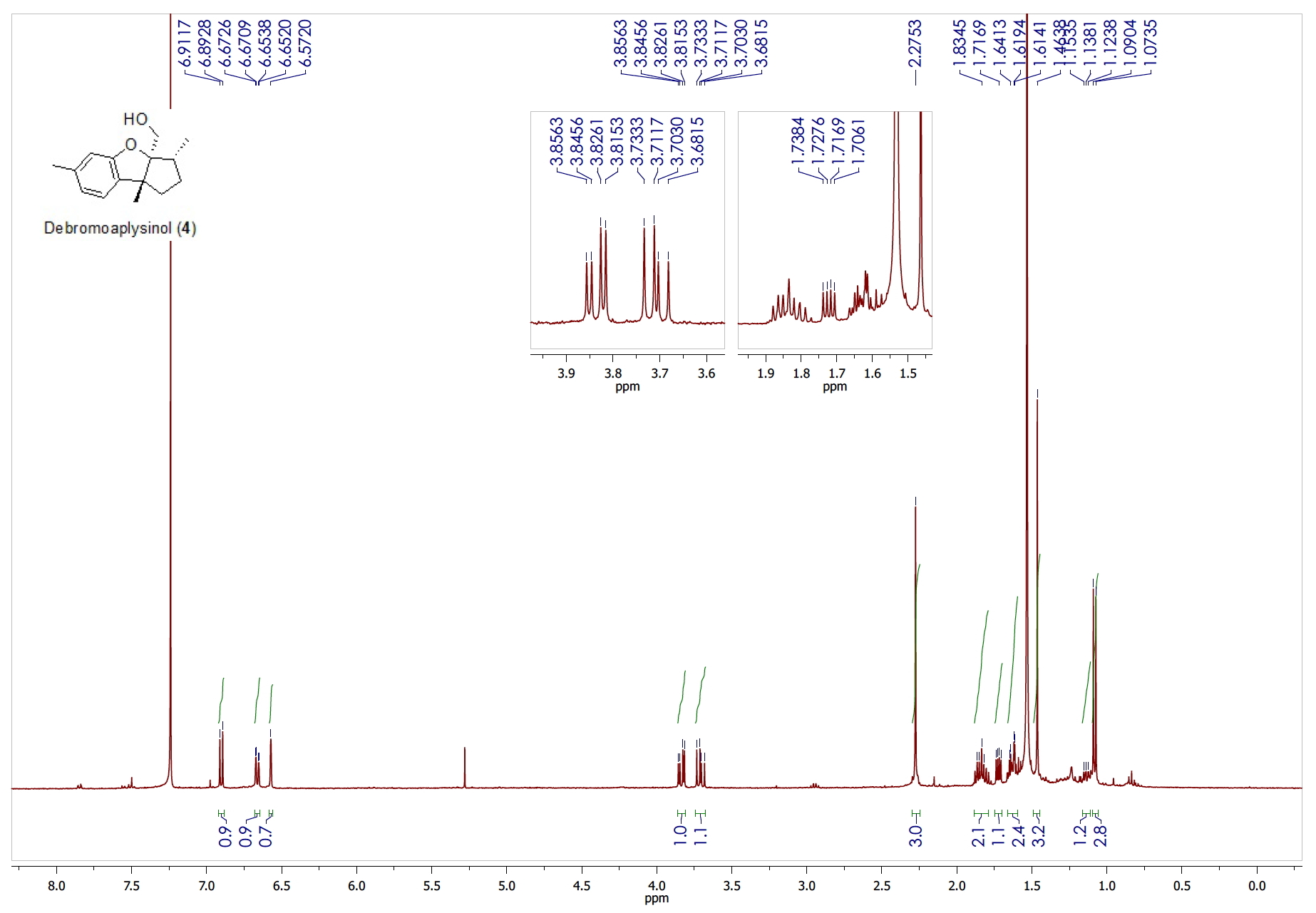


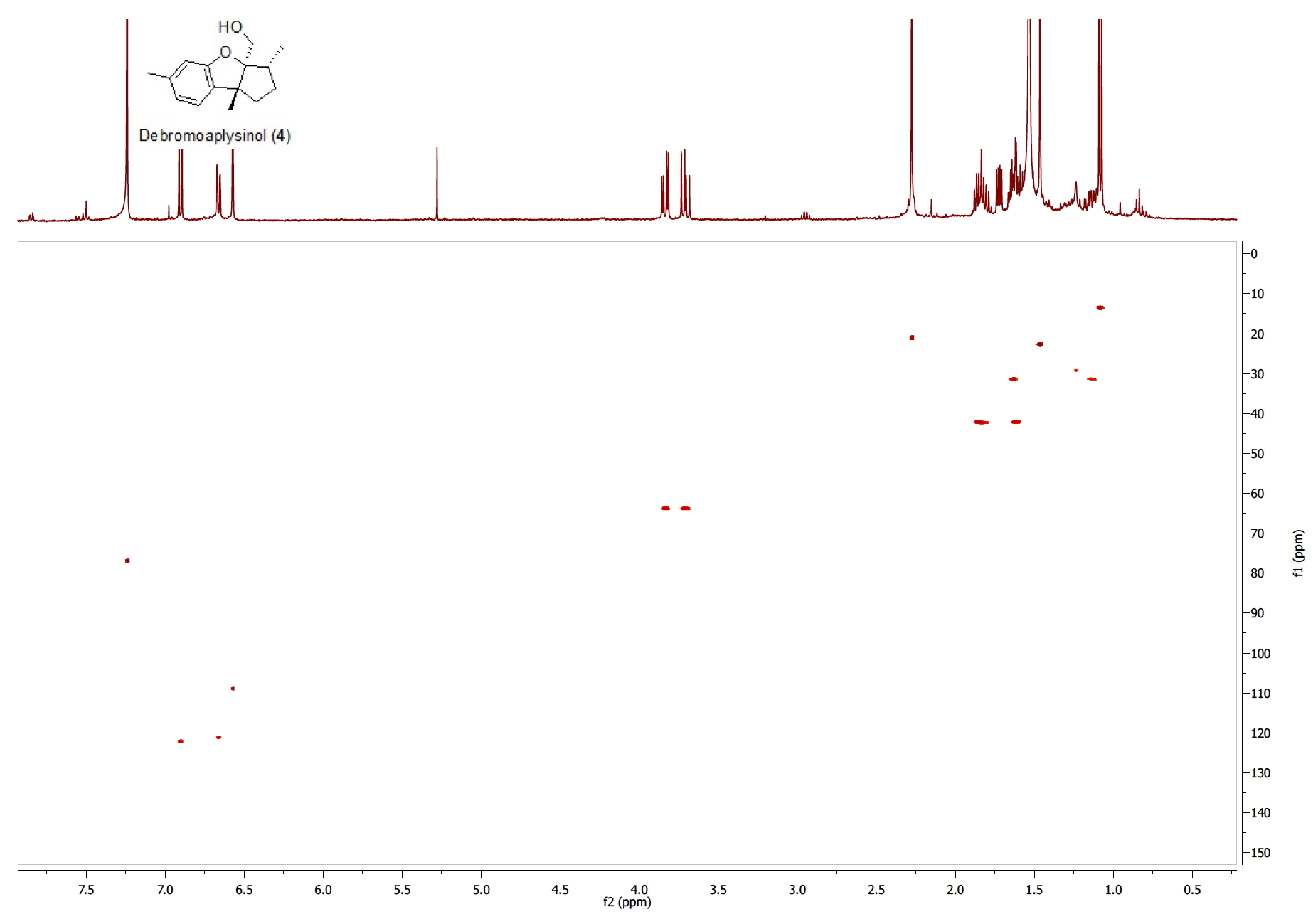


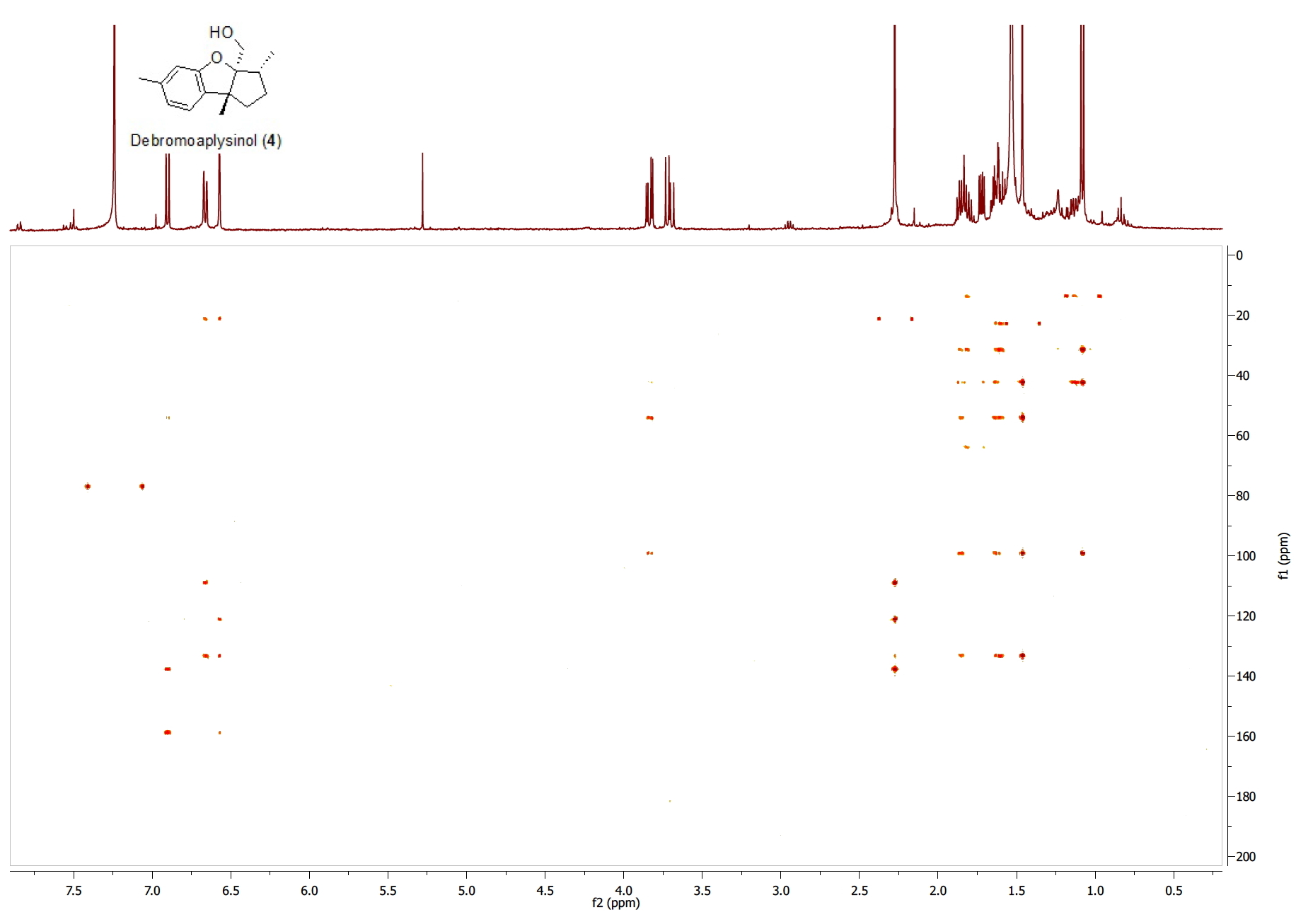


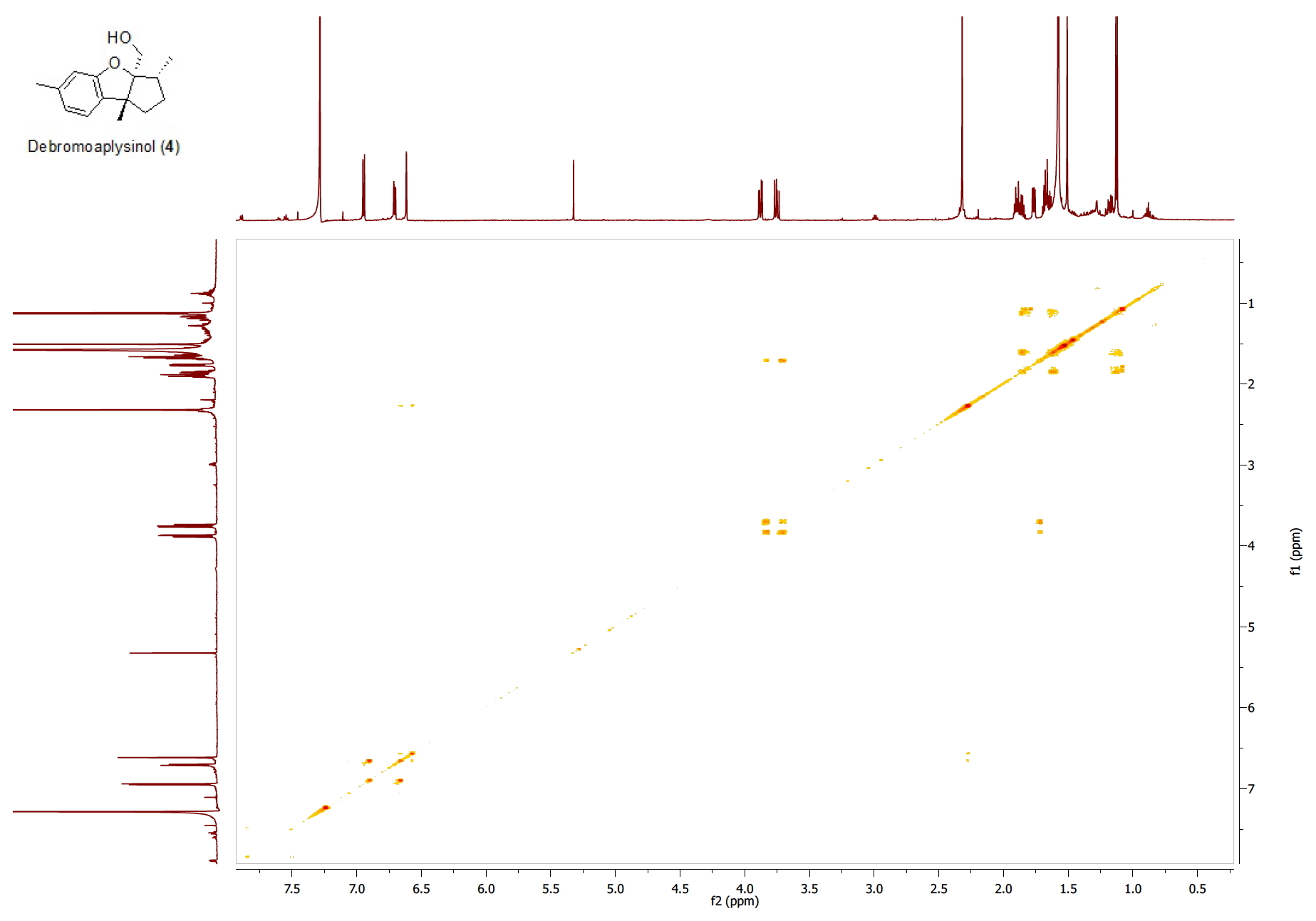


**NMR Spectra of 5.**


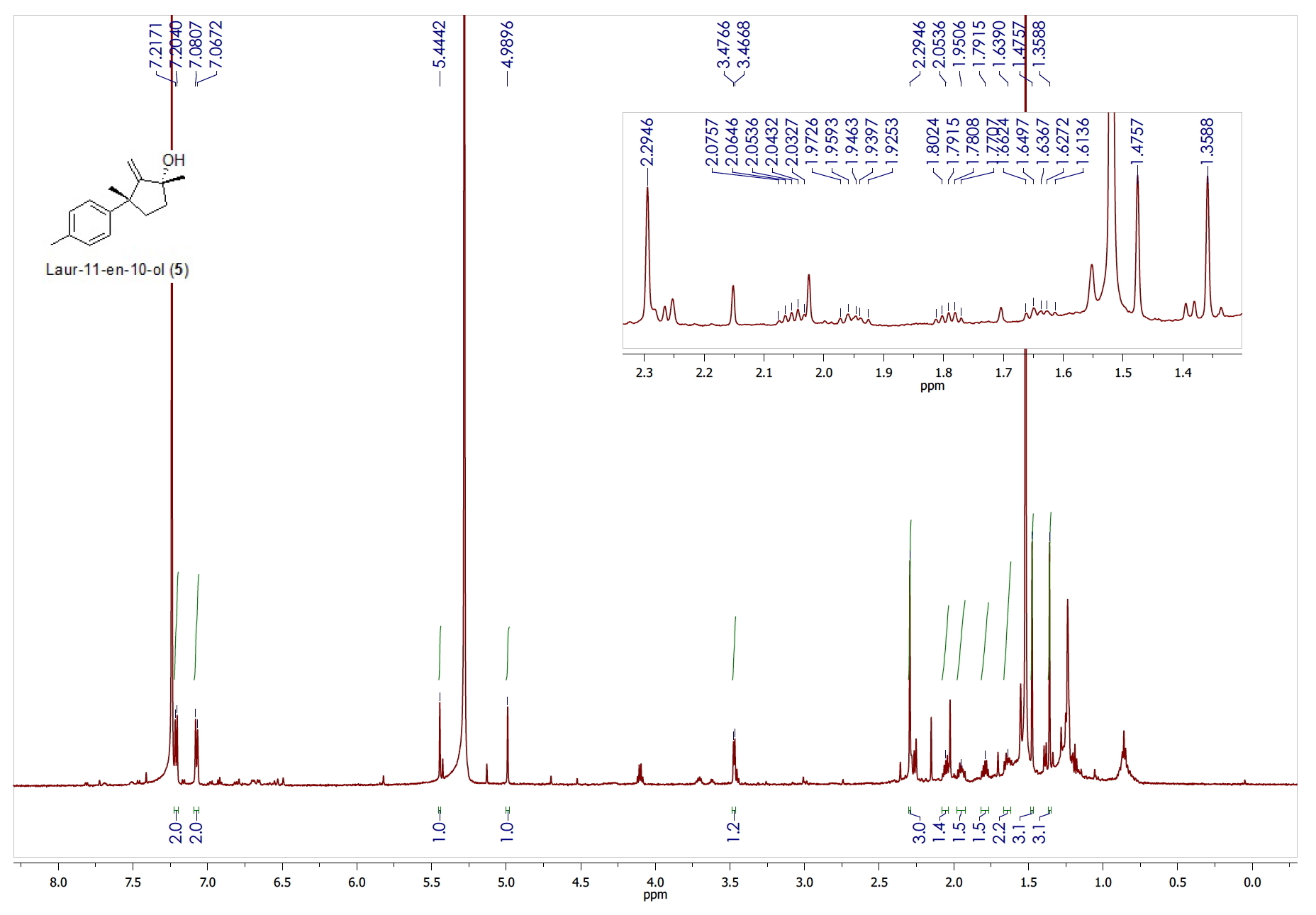


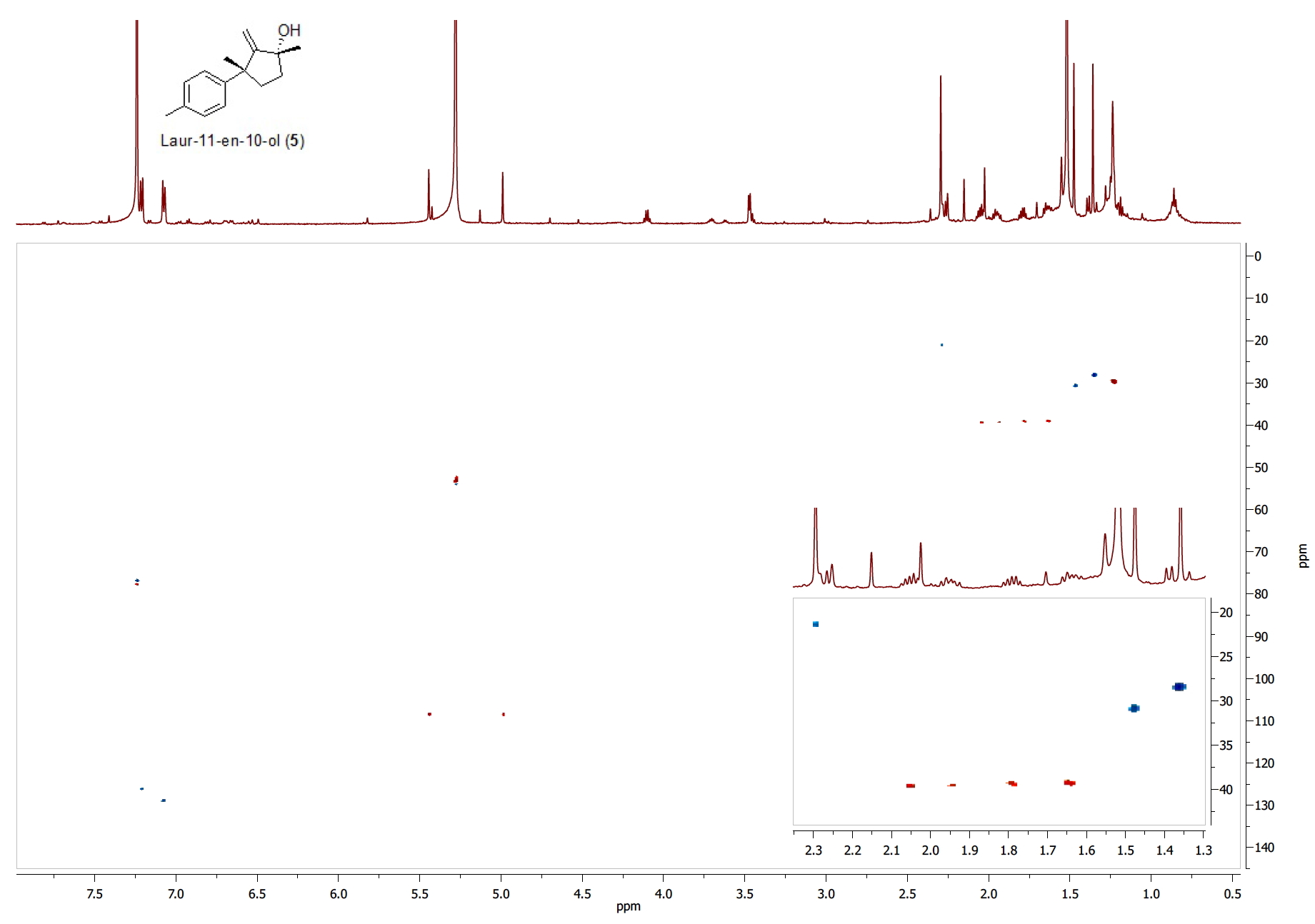


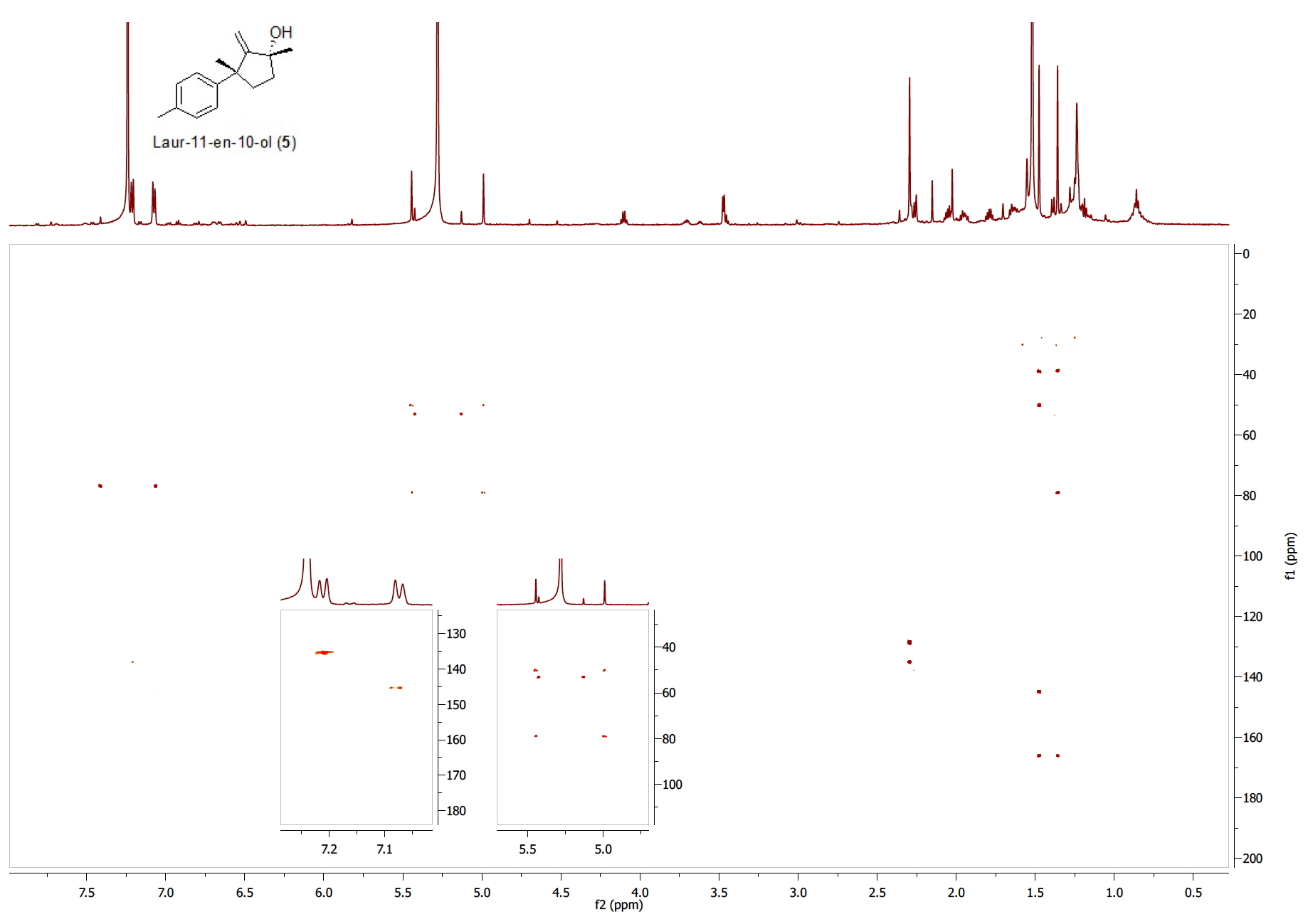


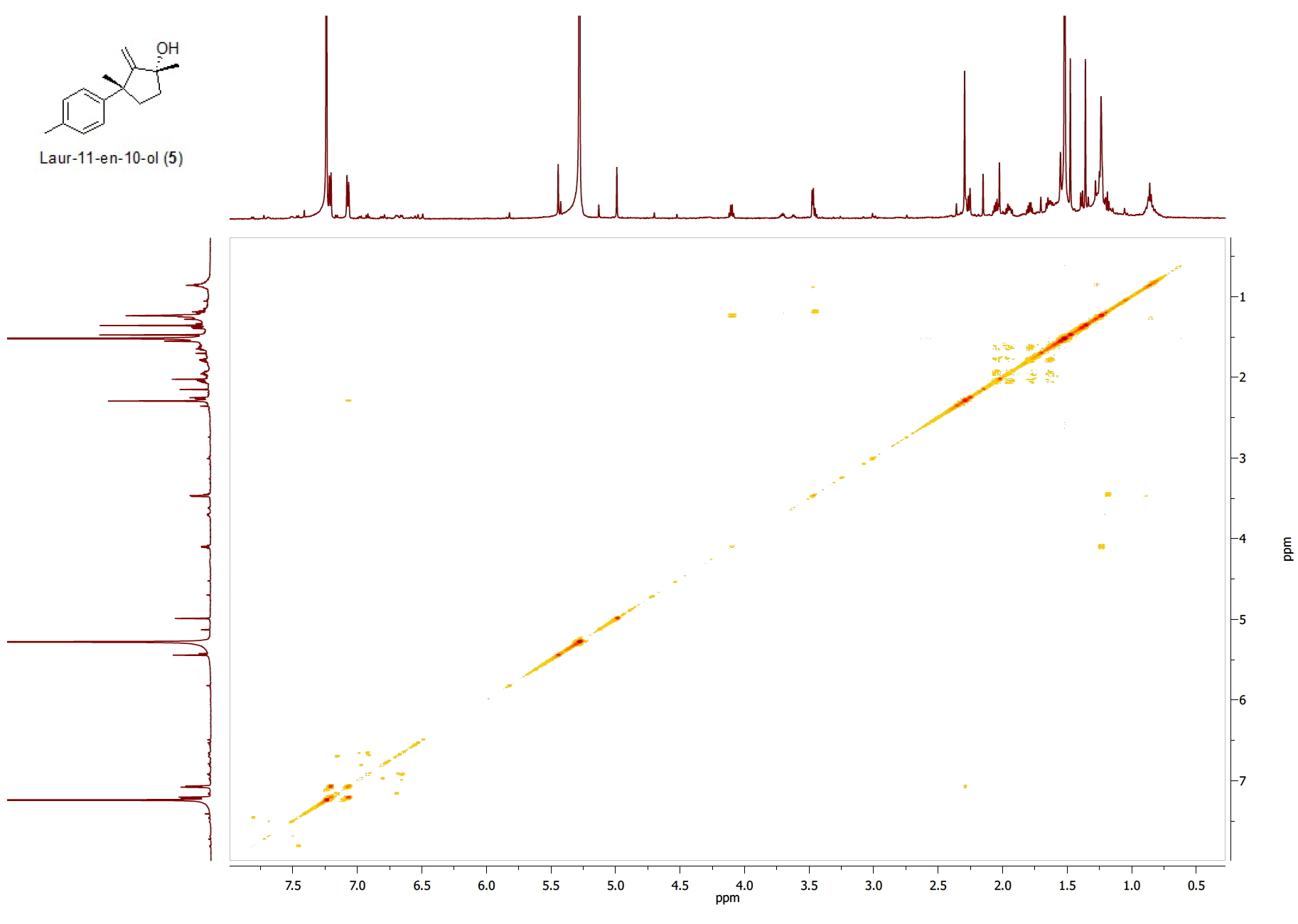


**NMR Spectra of 6 and 7.**


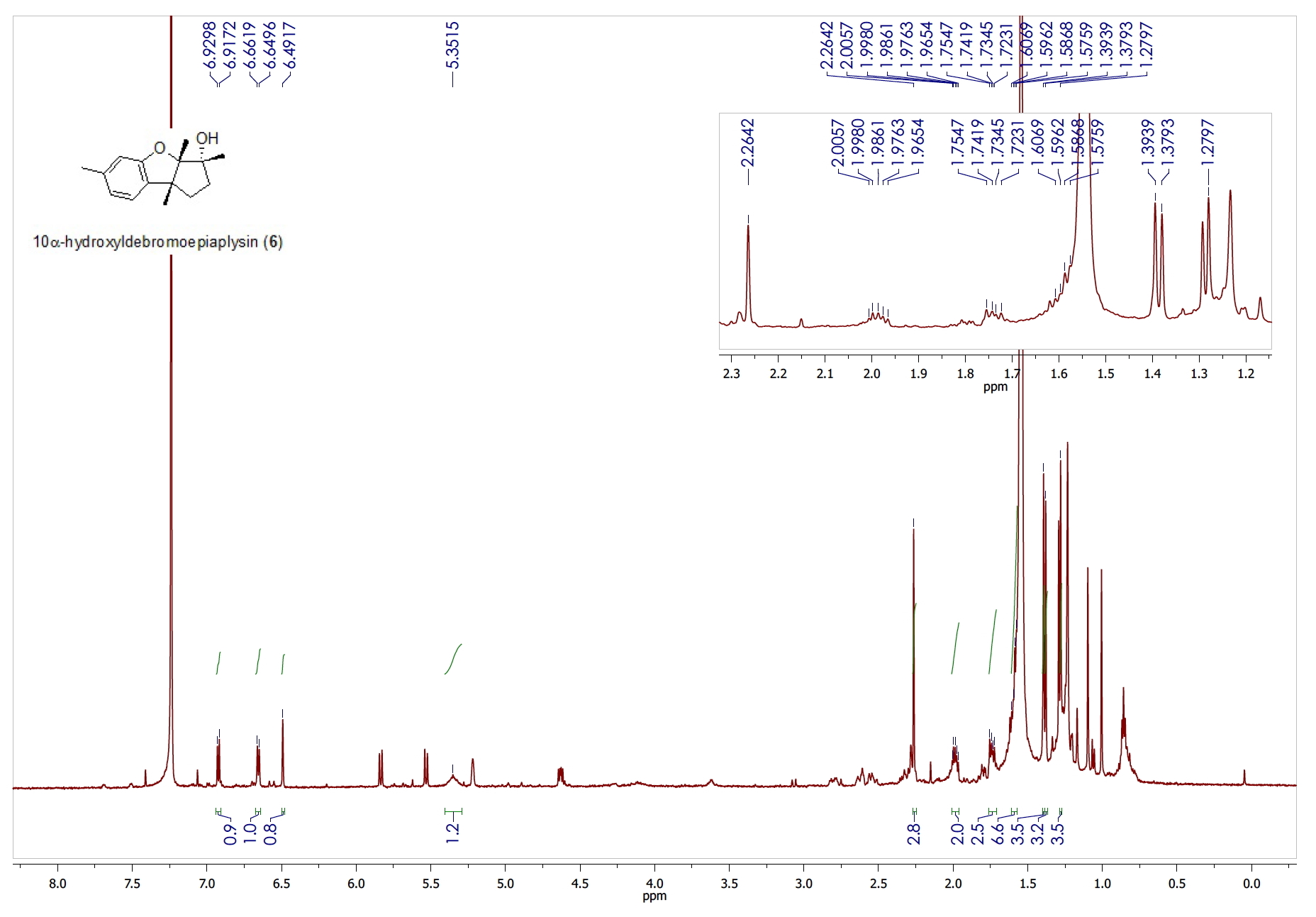


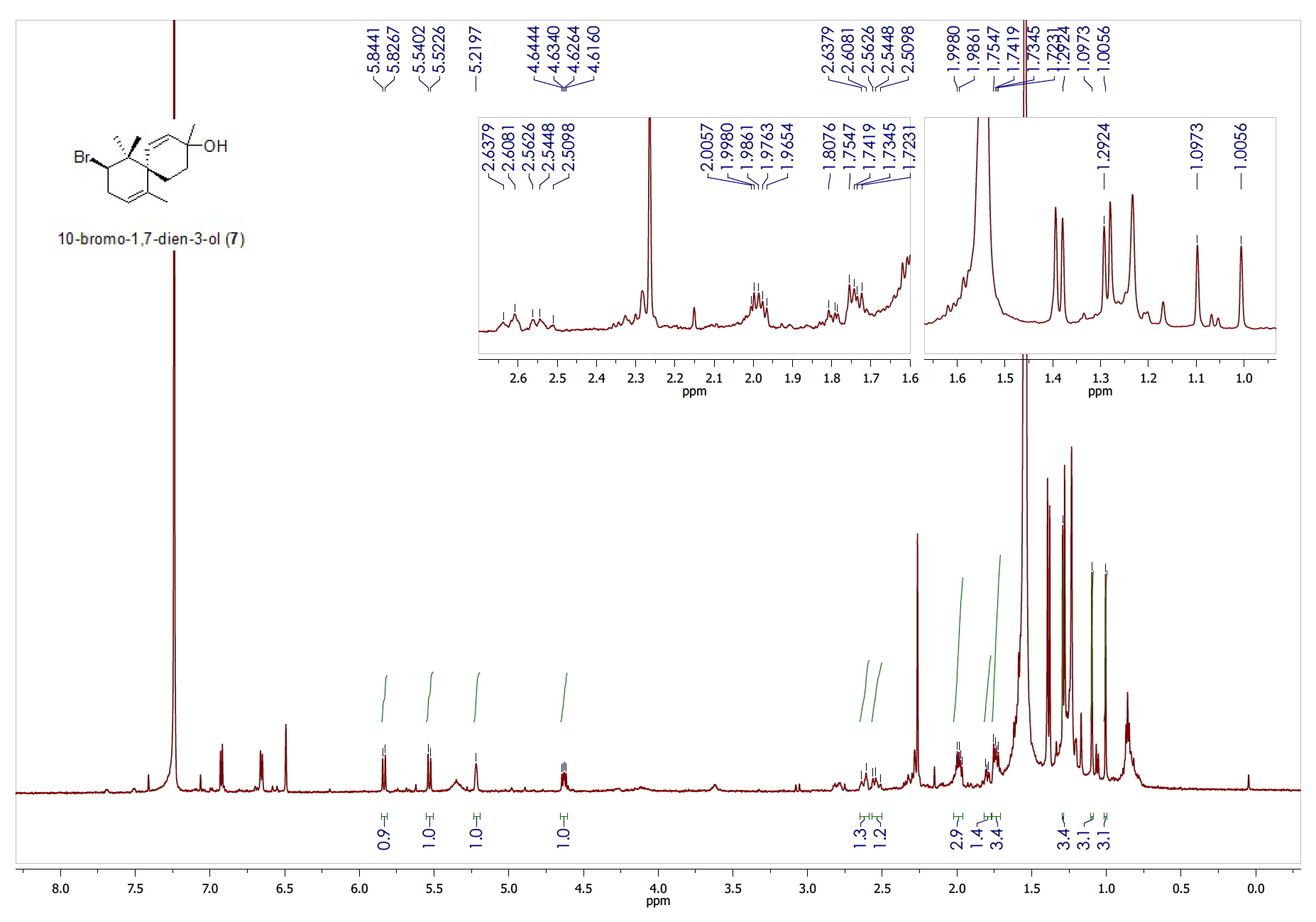


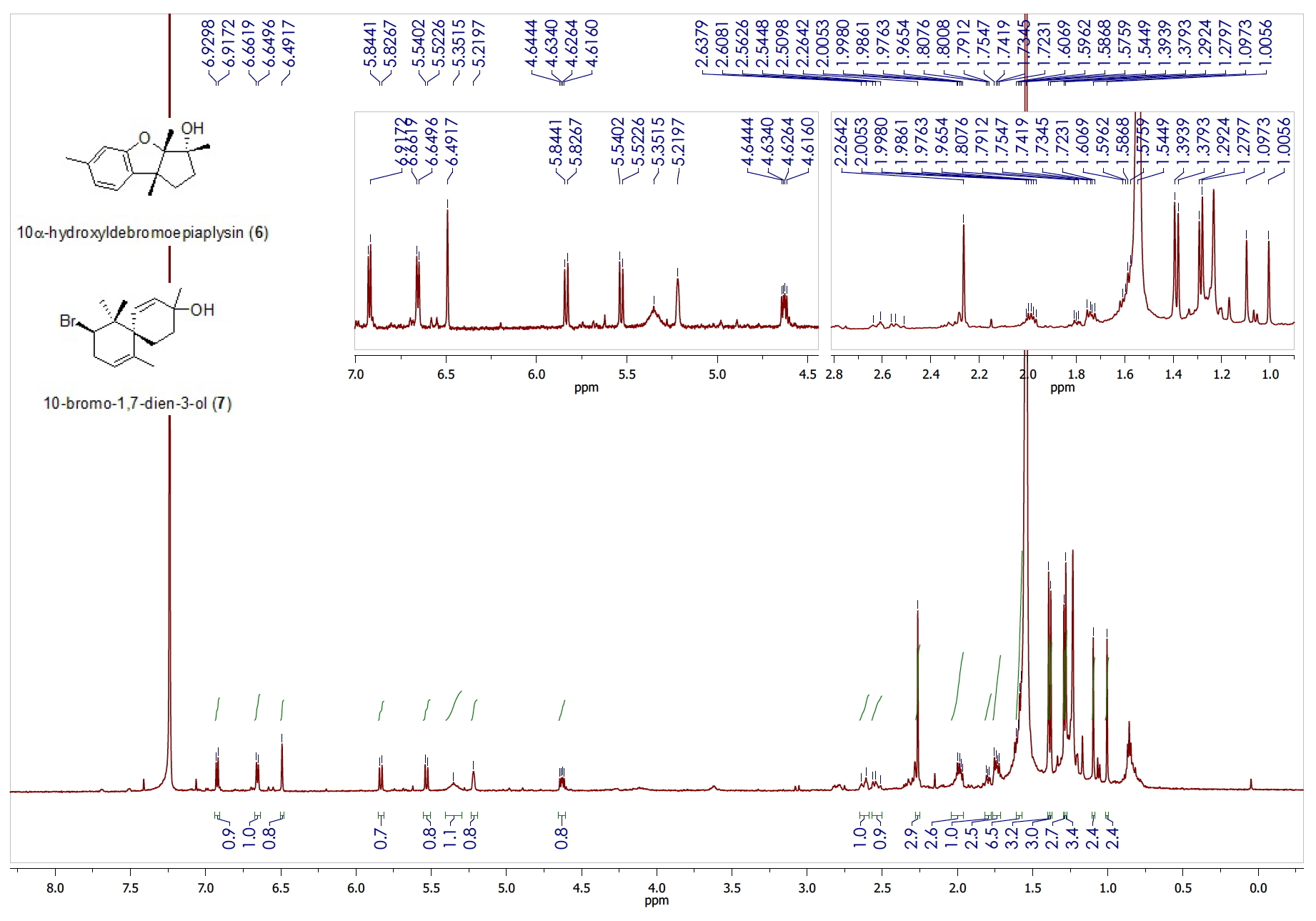


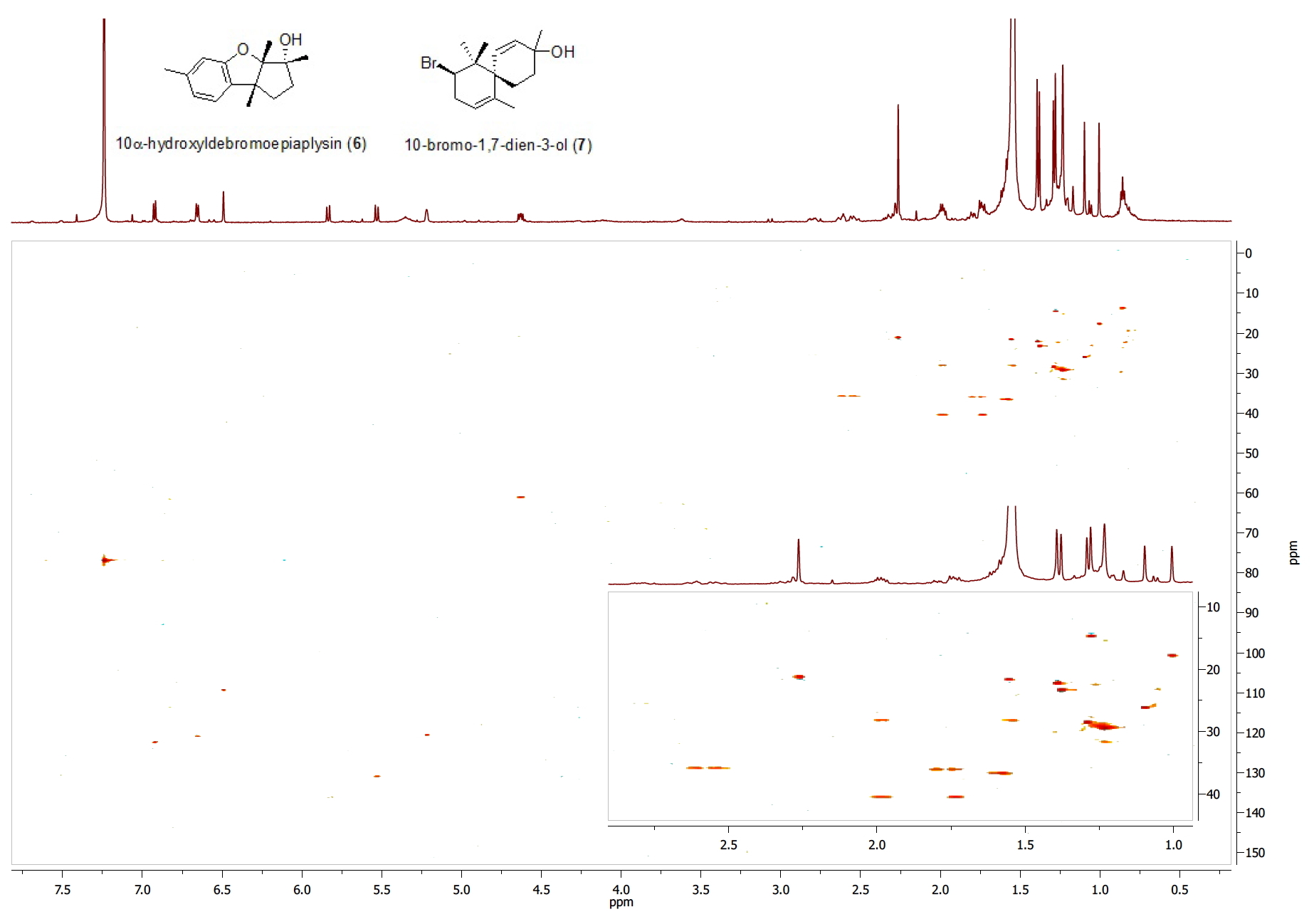


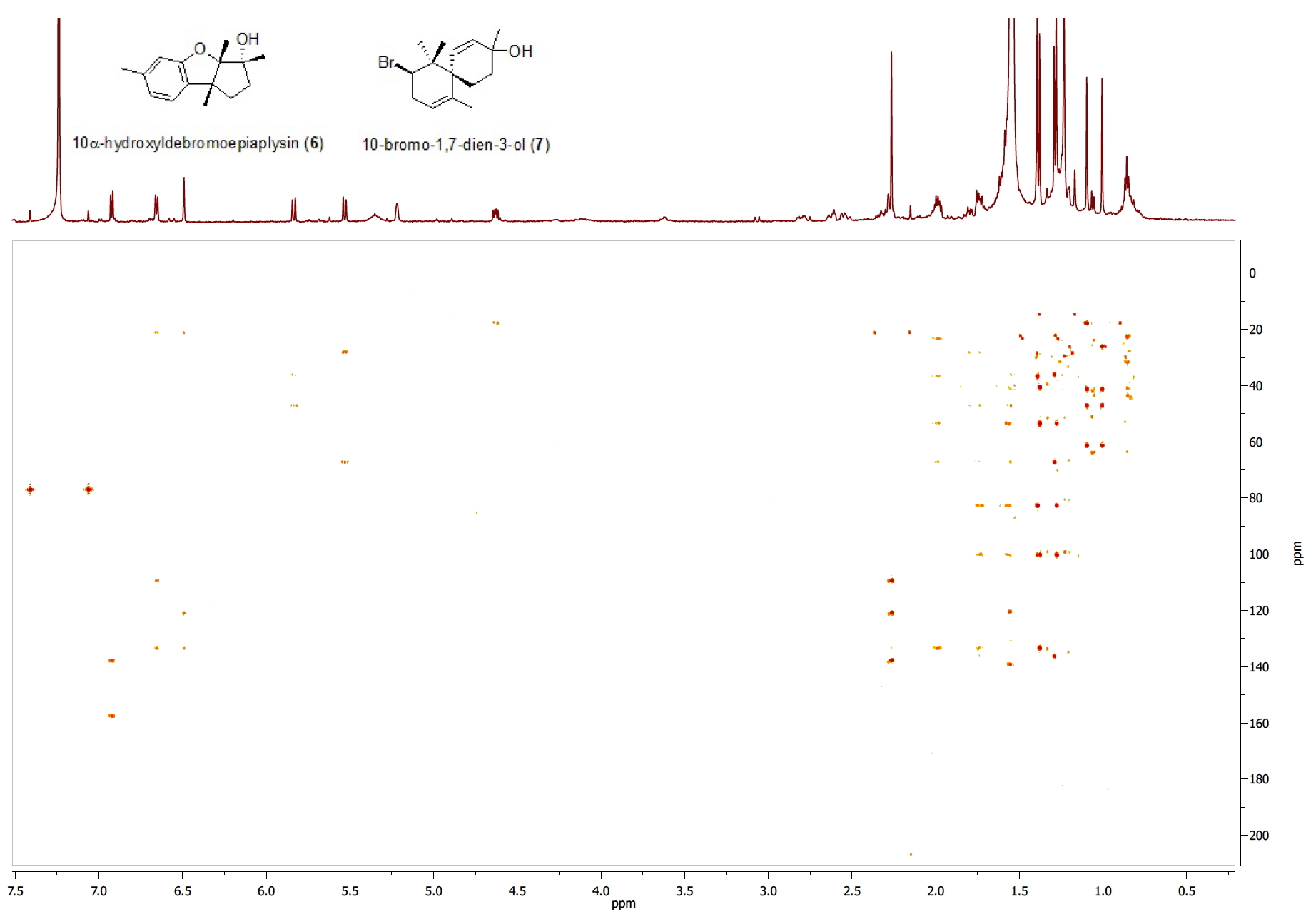


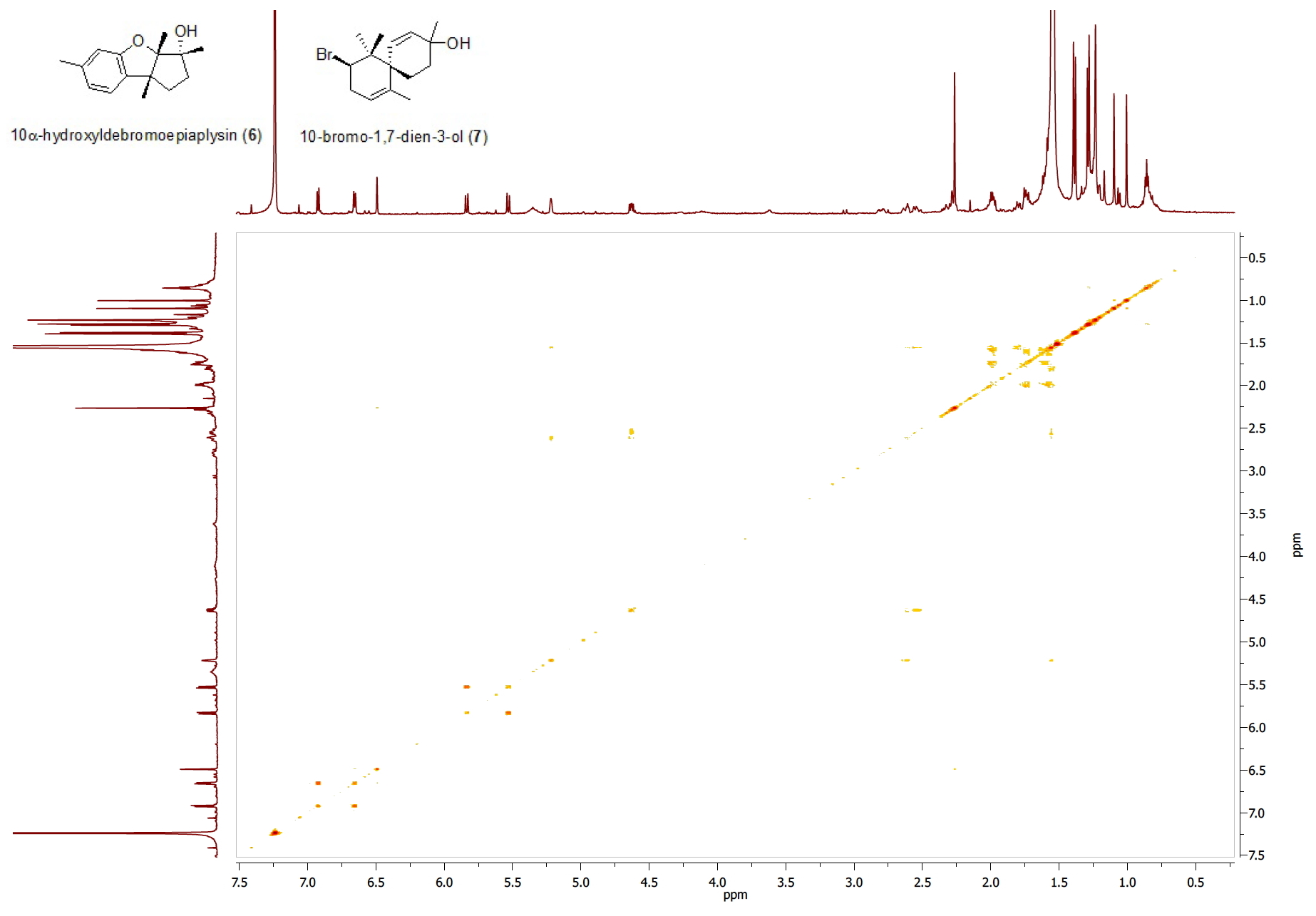

Supplement: Additional file 1: — Structural characterization. [file s13588-014-0008-8-S1.docx]
